# Sector-specific clean water scarcity and competition under global change

**DOI:** 10.1038/s41545-026-00608-0

**Published:** 2026-08-10

**Authors:** Gabriel A. Cárdenas Belleza, L. P. H. Rens van Beek, Marc F. P. Bierkens, Edward R. Jones, Michelle T. H. van Vliet

**Affiliations:** Department of Physical Geography, Utrecht University, Utrecht, The Netherlands

**Keywords:** Climate sciences, Engineering, Environmental sciences, Hydrology, Water resources

## Abstract

While the impact of future climate change on physical water availability has been extensively studied, the interplay with water quality and the associated impact on water scarcity—particularly the ensuing competition among sectors (domestic, irrigation, livestock, manufacturing, and energy) for clean water—is less well understood. Here we employed a globally applicable modelling framework to assess future cross-sectoral clean water gaps arising from competition for limited clean water resources, explicitly accounting for sector-specific water quantity and quality requirements under global change. Our results show that deteriorating water quality and insufficient water quantity will severely exacerbate future water gaps, exposing nearly 67% of the world’s population to severe clean water gaps by 2100. As a result, cross-sectoral competition for clean water will greatly intensify, with disproportional impacts on the manufacturing and thermoelectric sectors, each facing a 20% increase in water gaps relative to their demands, primarily related to rising water temperatures. Overall, our findings underscore the need for integrated water management strategies that address both water quantity and quality to mitigate growing competition for clean water.

## Introduction

Water plays a critical role in supporting a wide range of human activities, including domestic use, irrigation, manufacturing, and energy generation, the viability of all of which depend on sufficient quantity and quality^1,2^. Currently, populations in diverse regions across the world experience severe water scarcity, with a multitude of impacts on their water-dependent sectoral activities^3–6^. Furthermore, projected increase in sectoral water demands driven by global population growth and economic development^7–9^, together with expected deterioration in future surface water quality^10–12^ further exacerbate the current water scarcity conditions across the world^13^.

Global-scale modelling studies of future water scarcity have consistently demonstrated increasing population exposed to water scarcity under different global change scenarios^13–16^. Moreover, research including the effect of water quality portray that present and future global population will be severely exposed to clean water scarcity^1,13,17^. Nevertheless, these studies do not address the role of sector-specific water quality requirements and the resulting cross-sectoral competition for clean water.

Here, we developed a new approach to assess future water scarcity for the main water use sectors (i.e., domestic, irrigation, livestock, manufacturing, and thermoelectric), considering sector-specific water quality requirements and sectoral competition for limited clean water resources at the global scale. We quantify the water scarcity using the water gap concept, defined as the unmet demand remaining after supply that leads to unsustainable water use^14,18^. We distinguish between the ‘*clean water gaps’* driven by quality constraints and the ‘*water gaps’* caused solely by limited availability. Our analysis incorporates both volumetric (clean) water gaps and their demand-normalized estimates―henceforth, relative (clean) water gaps―, facilitating intersectoral and regional comparisons. Our approach explicitly incorporates both water quantity and sectoral water quality requirements under climate change and socioeconomic developments, allowing for: (1) the identification of sector-specific impacts of clean water gaps; (2) the attribution of the roles that water quantity (i.e., insufficient water availability) and quality (i.e., unsuitable water quality) aspects play in the water gap dynamics; and (3) the assessment of cross-sectoral competition for scarce clean water resources.

To this end, we employed a modelling framework that integrates renewable water availability (i.e., surface water and groundwater) with surface water quality. These components are simulated using high-resolution, process-based global models: PCR-GLOBWB 2^19^ for hydrology and DynQual v1.0^20^ for surface water quality. Sectoral water use and allocation are dynamically assessed using QUAlloc v1.0^21^, which has been further developed for this assessment (for details and equations see Supplementary Text 1 to 3, as well as Supplementary Fig. 1 and 2). Our analysis covers the period of 2005-2100. Given the high computational demand of our modelling framework, we focus on socioeconomic and climate change projections following the Shared Socioeconomic Pathway SSP3 (regional rivalry) and Representative Concentration Pathway RCP7.0. This scenario reflects a world characterized by strong population growth, regional rivalry, relatively high greenhouse gas emissions and limited adaptation to climate change, and has been argued to be closely reflective of the trajectory the world is currently following^22,23^. To account for uncertainties in the structure and parameterizations of Global Climate Models (GCMs) we used bias-corrected outputs of the five primary GCMs of the ISIMIP 3b protocol: gfdl-esm4, ipsl-cm6a-lr, mpi-esm1-2-hr, mri-esm2-0, ukesm1-0-ll^24^ (see Methods section).

## Results

### Water gap drivers across water use sectors

**Fig. 1:**
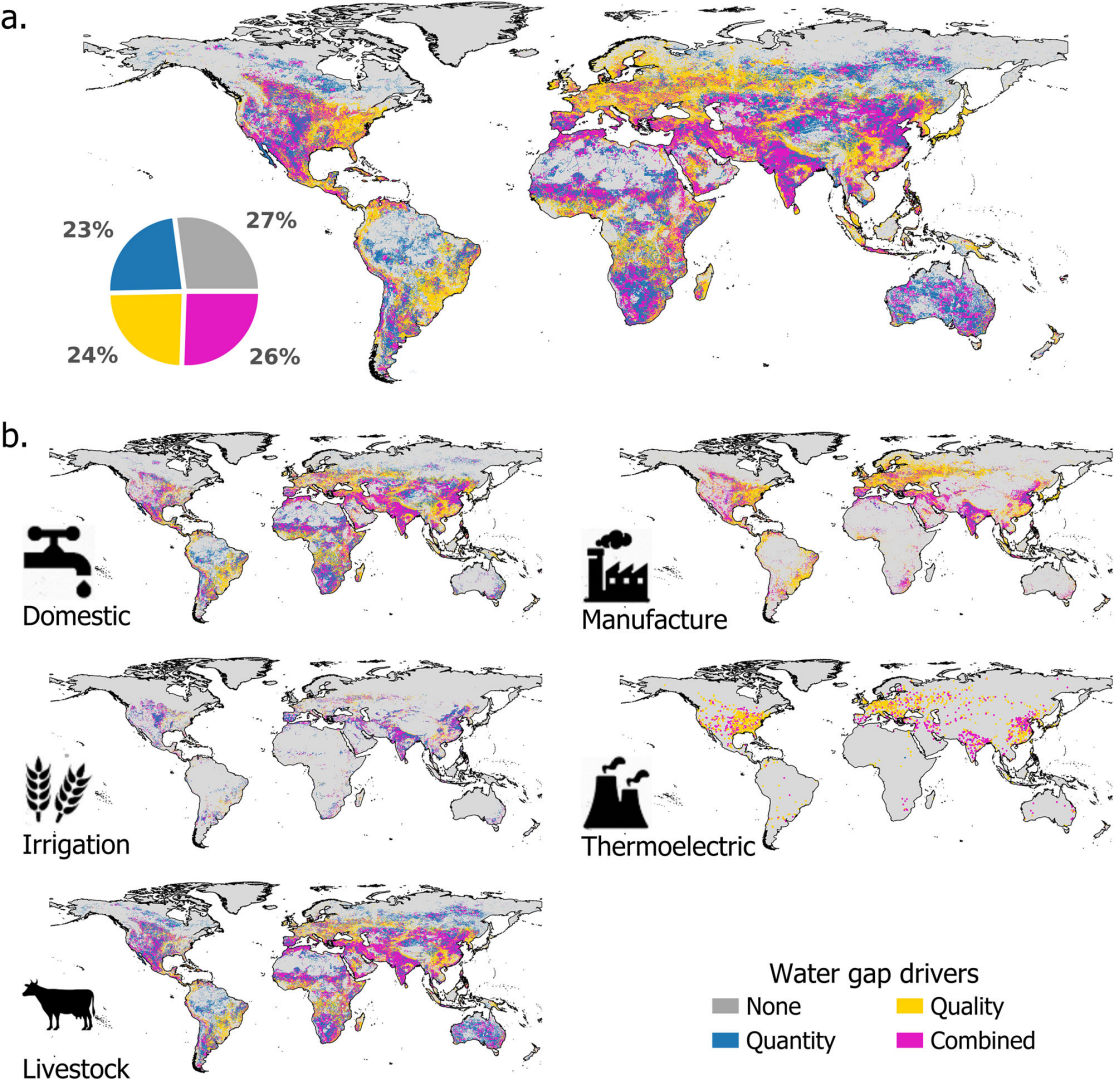
Future water gap drivers at the end of the century (2081–2100). **a** Total water gap (i.e., sum of all sectoral water gaps), where pie chart indicates percentage of inhabited areas affected by a specific driver; **b** per sector (i.e., domestic, irrigation, livestock, manufacturing, and thermoelectric). *Quantity driven* indicates insufficient water availability to meet sectoral water quantity demands (no water quality constraints), *quality driven* indicates exceedance of water quality thresholds for sectoral use (sufficient water availability), and their *combined effect* indicates that both water quantity and quality issues drive the water gap. Data employed correspond to the ensemble mean of the simulations performed using bias-corrected output of five GCMs for the global change scenario SSP3-RCP7.0. Displayed drivers are the most recurrent annual water gap driver over the period 2081–2100 over each grid-cell.

Our results reveal a substantial expansion in inhabited areas—defined as locations with human water demands— affected by water gaps by the end of the century (2081–2100), with a projected increase of 28% compared to the reference period (2005–2020) (Fig. 1a and Supplementary Fig. 3a). In this context, the combined impact of unsuitable water quality and insufficient water quantity emerges as the predominant driving mechanism of the future water gaps, affecting 26% of inhabited areas, primarily in densely populated regions of South Asia, Sub-Saharan Africa, the Middle East, and eastern Pacific Asia (Fig. 1a). These spatial patterns are also observed in the domestic sector (Fig. 1b and Supplementary Fig. 4b), with Sub-Saharan Africa and South Asia in particular being associated with limited expansions in water treatment capacities relative to the projected population growth^25^.

The impact of water quality deterioration constituted the second most significant driver of projected water gaps, expanding its spatial coverage by 12% over the century and affecting up to 24% of inhabited areas. This shift highlights a pronounced deterioration in water quality over time, likely resulting from increased water pollution from human activities associated with population growth. Additionally, climate variations may create conditions that further exacerbate this decline in water quality (e.g., increased water temperatures) and a limited dilution capacity under changing flow regimes^13^.

The manufacturing sector experiences the largest increase in inhabited areas affected by water quality deterioration, with a 24% rise projected by the end of the century (Supplementary Fig. 4b). This condition is primarily driven by increasing water temperatures, resulting unsuitable conditions for cooling water use purposes. As a result, only 5% of manufacturing water-consuming areas are projected to remain unaffected by water gaps. The eastern United States, Europe, and central Asia show the largest expansions in areas affected by unsuitable water quality as the main driving mechanism for water gaps (Fig. 1b).

Water gaps driven exclusively by insufficient water quantity (Fig. 1b) predominantly affect the irrigation sector in major agricultural regions, often referred to as global “food bowls”, like the Great Plains (US), Spain, and the Yangtze basin (China). These patterns likely reflect the increasing food demand associated with population growth. However, this driver also emerges in regions with low population density or limited water availability (e.g., central Australia and northern Canada) (Fig. 1a) where water scarcity is mostly associated with the livestock sector, which accounts for 1% of global water demand^9,26^. In these areas, livestock water requirements are minimal, with a median of 0.25 m^3^/day at a 5 arcmin grid-cell level, resulting in corresponding low water gaps, but also implying a high sensitivity to water availability changes.

### Clean water gaps over time

**Fig. 2:**
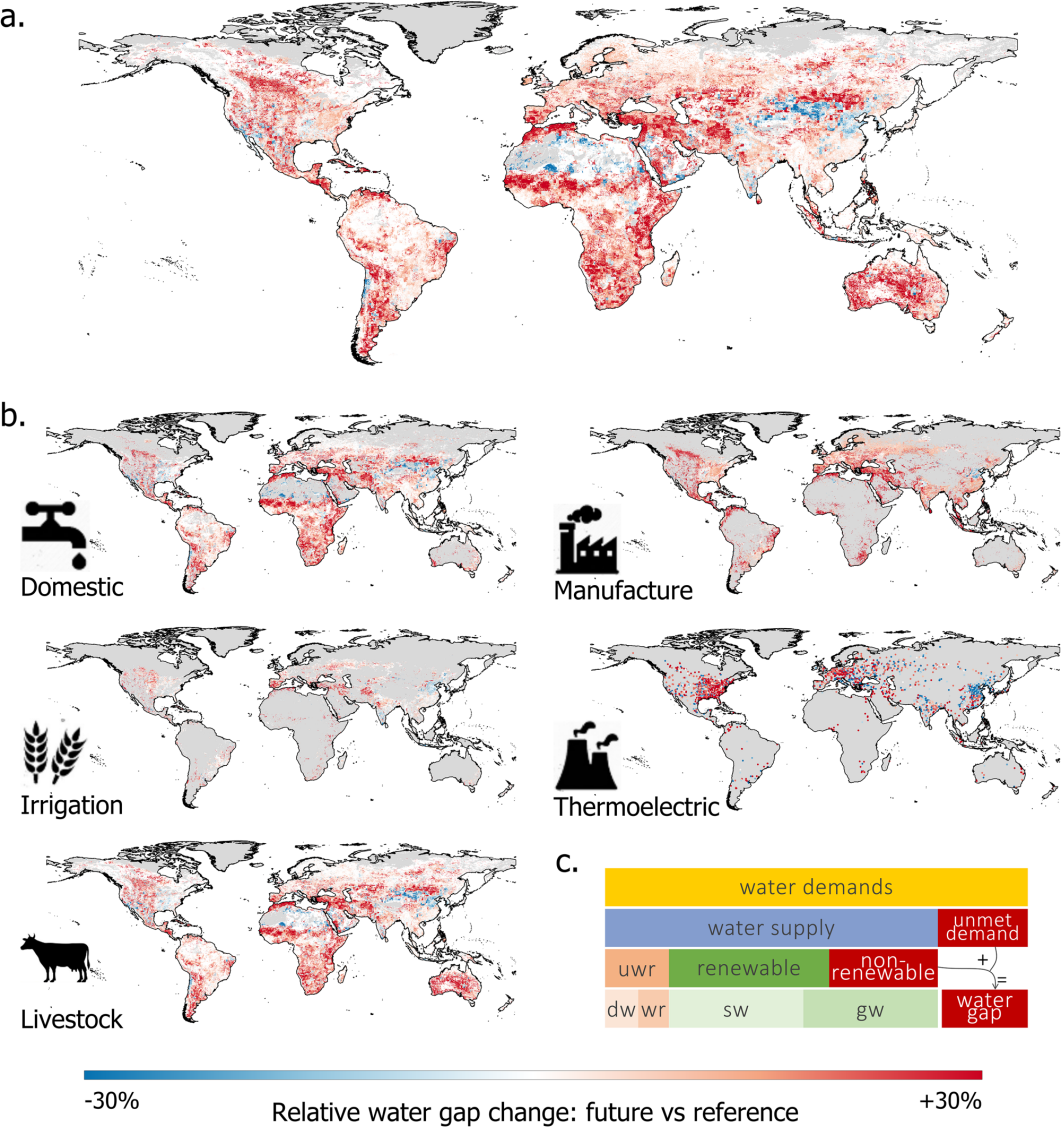
Change in the relative clean water gaps for future (2081–2100) versus reference period (2005–2020). *Relative water gap change* (in percentage points) results from the difference between the mean relative clean water gap over the end-of-century period (2081–2100) minus the mean relative clean water gap over the reference period (2005–2020). Data employed correspond to the ensemble mean of the simulations using the bias-corrected output of five GCMs for the global change scenario SSP3-RCP7.0 when sectoral-specific quality requirements are considered. **a** total water gap (i.e., sum of all sectoral water gaps), **b** per sector (i.e., domestic, irrigation, livestock, manufacturing, and thermoelectric), **c** conceptualization of potential water sources and water gap estimation; where: *uwr* = unconventional water resources, *dw* = desalinated water, *wr* = wastewater reuse, *sw* = surface water, *gw* = groundwater.

**Fig. 3:**
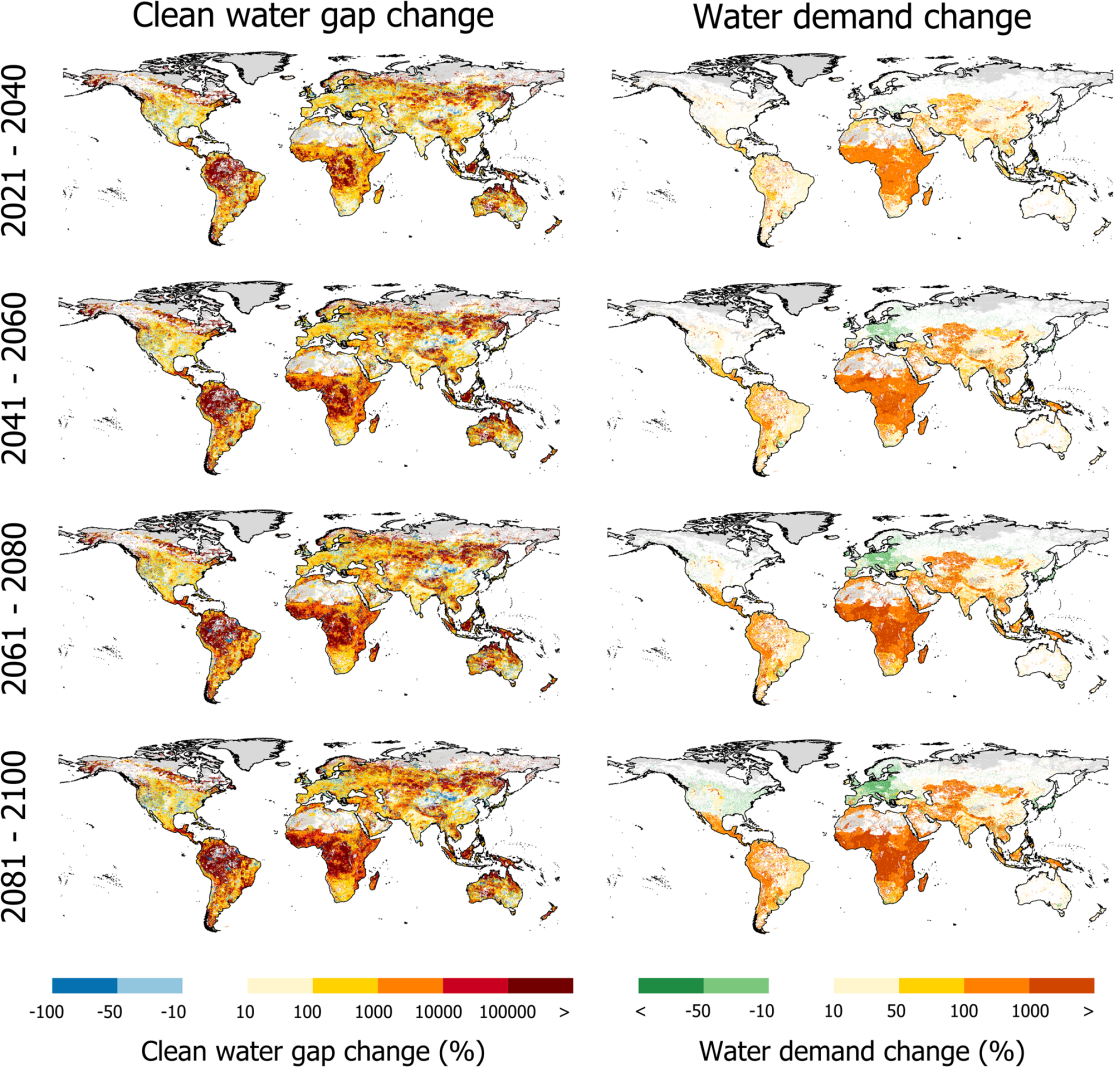
Change in the clean water gap and the water demand: future periods (2021–2100, every 20 years) relative to the reference period (2005–2020). Left column displays the volumetric total clean water gap (i.e., sum of water gaps from all sectors) for the ensemble of the bias-corrected output of five GCMs for the global change scenario SSP3-RCP7.0 when sectoral specific water quality requirements are considered. Right column displays the change in future total water demands (i.e., sum of sectoral water demands from all sectors).

Our study differentiates between *water gaps*, which result from insufficient water availability in terms of its quantity, and *clean water gaps*, which additionally account for constraints related to water quality. We project an overall increase in the relative clean water gap (i.e., clean water gap divided by water demand) at the end of the century (2081–2100) compared to the conditions during the reference period (2005–2020) for all water use sectors (Fig. 2). The largest increases in relative clean water gaps are in the North Africa & Middle East geographic region (median increase of 20% over areas where clean water gap increases) followed by Sub-Saharan Africa ( + 14%), southern East & Pacific Asia ( + 10%) and Latin America & Caribbean ( + 9%). Moreover, while the North Africa & Middle East region is projected to experience the largest clean water gaps in the domestic sector, with median increase of 20%, Sub-Saharan Africa shows the highest impact for the manufacturing sector, with a projected median increase of 31% in future clean water gaps. In both regions, clean water gaps are primarily driven by the combined effect of deteriorated water quality status and insufficient water availability (Fig. 1b) mostly resulting from increasing water demands (Fig. 3).

The irrigation sector shows an increase in future clean water gaps in locations commonly referred as “food bowls”, like California, Spain and Türkiye, as well as in central Asia (i.e., Afghanistan) and the Near East (Fig. 2b). These increasing clean water gaps for irrigation are mainly related to the combined effect of poor water quality and insufficient water availability. Likewise, the increase in projected clean water gap for the thermoelectric sector is mainly due to the increase in surface water temperature. Nonetheless, regions like Pacific China present a decrease in future thermoelectric clean water gaps (Fig. 2b). This is related to an increase in water availability for cooling purposes as the result of cross-sectoral competition for clean water: decreased levels of water available for other sectors due to unsuitable quality conditions, increases the available water for thermoelectric cooling.

**Fig. 4:**
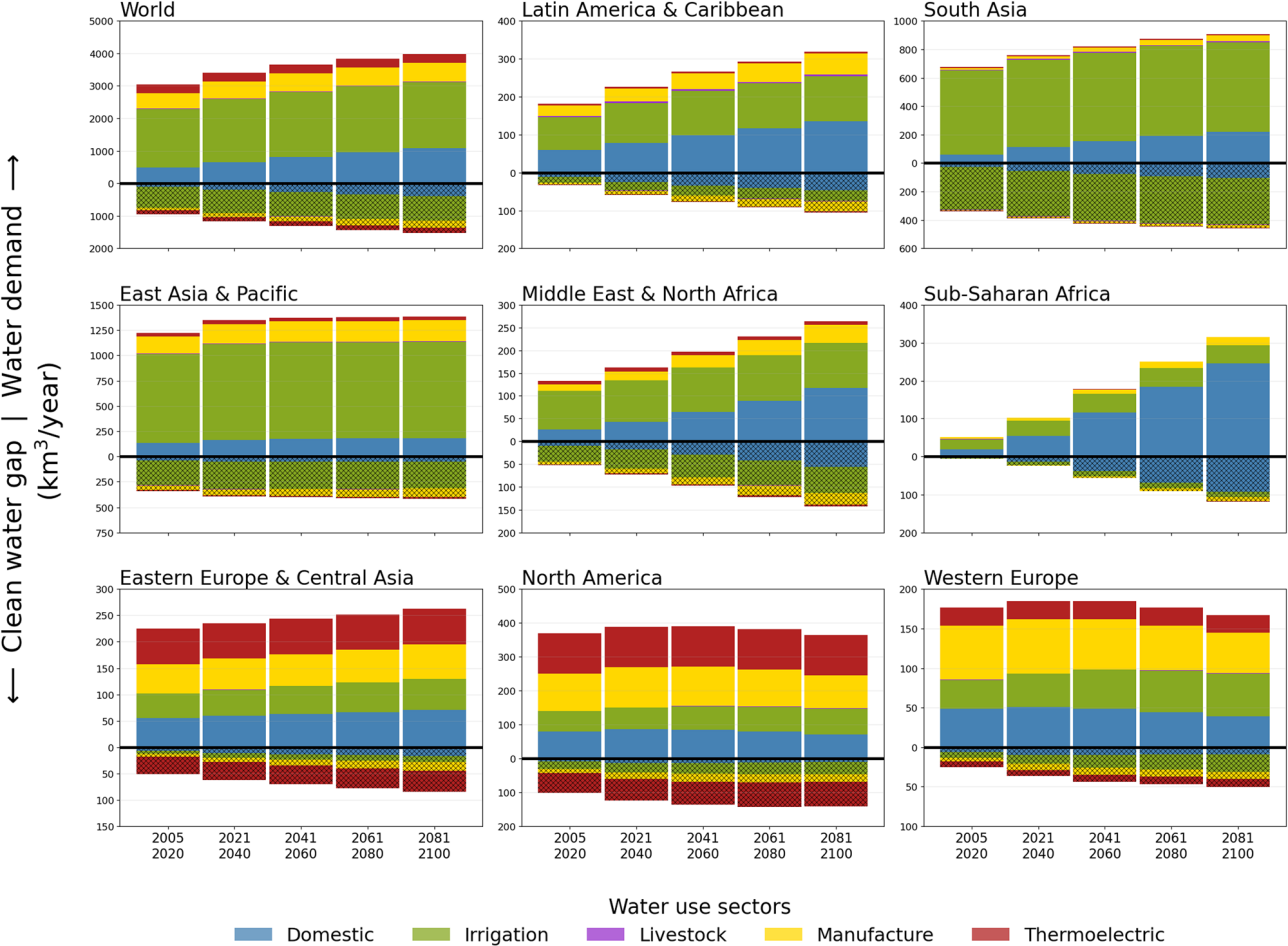
Comparison of sectoral water demands and clean water gap over time for the period 2005–2100 by geographic region, for the domestic, irrigation, livestock, manufacturing, and thermoelectric sectors. Results are displayed as a stacked bars chart with water demand (upper y-axis) and clean water gap (inverted y-axis), aggregated to the annual scale and averaged every 20-year time-slices. Data employed correspond to the ensemble mean of simulations using the bias-corrected output of five GCMs for the global change scenario SSP3-RCP7.0 when sectoral-specific water quality requirements are considered. Due to the low magnitudes of livestock clean water gap in comparison to the other sectors, these are hardly visible in the graphs.

Our results also identify regions where the clean water gap is projected to decrease by the end of the century, such as southern United States (Fig. 2a). This trend is primarily attributed to projected reductions in total water demand (Fig. 3, grid-cells in green), largely driven by the demographic trends (e.g., ageing population, slower population growth) in SSP3 that reduce water demands from domestic sector over the second half of the century (Fig. 4). Although this trend is also projected in Western Europe, this does not lead to a decrease in water gap over time, presumably due to lower clean water availability (Fig. 1 and Supplementary Fig. 5). Additional regions where clean water gaps are projected to decrease are arid zones like the Sahara and northeastern China. These areas naturally exhibit low water demand (a median of 150 m^3^/day at a 5 arcmin grid-cell level), making them particularly sensitive to changes in water availability and, therefore, highly uncertain.

Overall, Latin America & Caribbean, the Middle East & North Africa, and Sub-Saharan Africa are the geographical regions that display the most notable increase in water demands, mainly attributable to the domestic sector (Figs. 3 and 4). Sub-Saharan Africa exhibits the largest relative increase (1225%), far exceeding the global average (120%), driven by rapid population growth. Nonetheless, this increasing pattern is also projected at the global level, turning the domestic sector into the sector with the largest rate of increasing clean water gap (+3.5 km^3^/year) over the 21st century (Fig. 4).

There is an overall global increase in the manufacturing clean water gap (average rate of +1.8 km^3^/year) over the century (Fig. 4), attributable to a combination progressively increasing in manufacturing water demand and the decreasing availability of clean water. East Asia & Pacific is the most affected region with a future clean water gap of 88 km^3^ annually. Also, the Middle East & North Africa region displays a noted increase in the manufacturing relative clean water gap: above 30% over 95 years (2005–2100) (Supplementary Fig. 5). Similarly, the regions projected to show the largest increases in the thermoelectric relative clean water gap are Sub-Saharan Africa (~51%/95 years) and the Middle East & North Africa (~29%/95 years), mainly caused by the increase in the temperature of the water used for cooling purposes.

The South Asia and East Asia & Pacific regions are the largest irrigation water users, accounting for about 78% global irrigation water demands, presenting proportionally high relative clean water gaps for irrigation (average of ~52% and ~28%, respectively) consistently over time (Fig. 4 and Supplementary Fig. 5). Conversely, regions like North America, Western Europe, Middle East & North Africa, and Sub-Saharan Africa are more severely affected by global change with an increase of approximately 20% each region of their irrigation relative clean water gaps over the 21^st^ century.

**Fig. 5:**
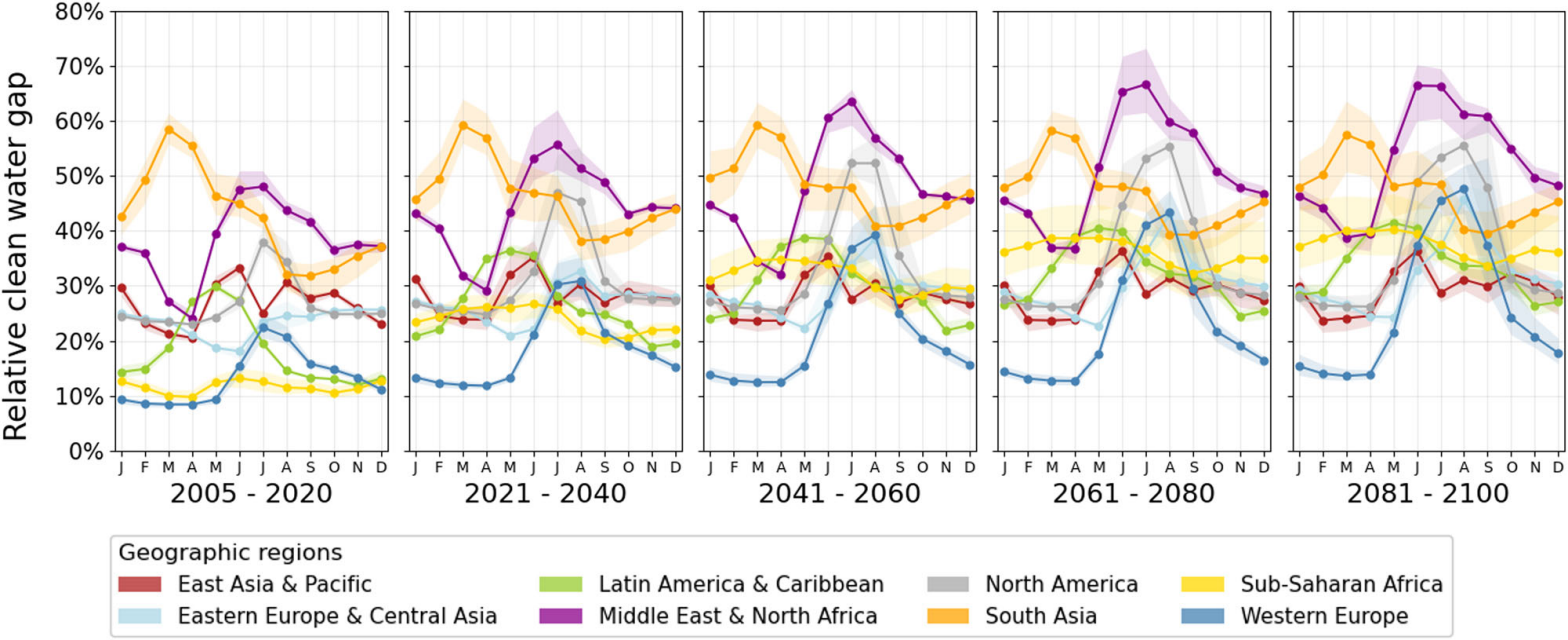
Seasonality of relative clean water gap for all sectors together per geographic region. Results are the multi-annual monthly averages every 20 years. Displayed results correspond to the ensemble mean (solid line) and the range of variation of their corresponding bias-corrected output of five GCMs (coloured shades) for the global change scenario SSP3-RCP7.0 when sectoral-specific quality requirements are considered.

Our results show that relative clean water gaps present a strong seasonality (Fig. 5), in which warmer months are associated with higher total clean water gaps, which are related to the increase in water demands and the deterioration of the water quality (i.e., increased temperature and increased salinity, organic, and pathogen pollution). Furthermore, these seasonal increases in total relative clean water gap show increasingly larger peaks over time for the northernmost regions: Western Europe (annual variation range: ~14% to ~34%), North America (~15%) and Eastern Europe & Central Asia (~14%) (Fig. 5). Regions like South Asia and Latin America & Caribbean have relatively smaller seasonal variations but also show distinct incrementally increasing clean water gaps over time.

### Water quality impacts on clean water gap

**Fig. 6:**
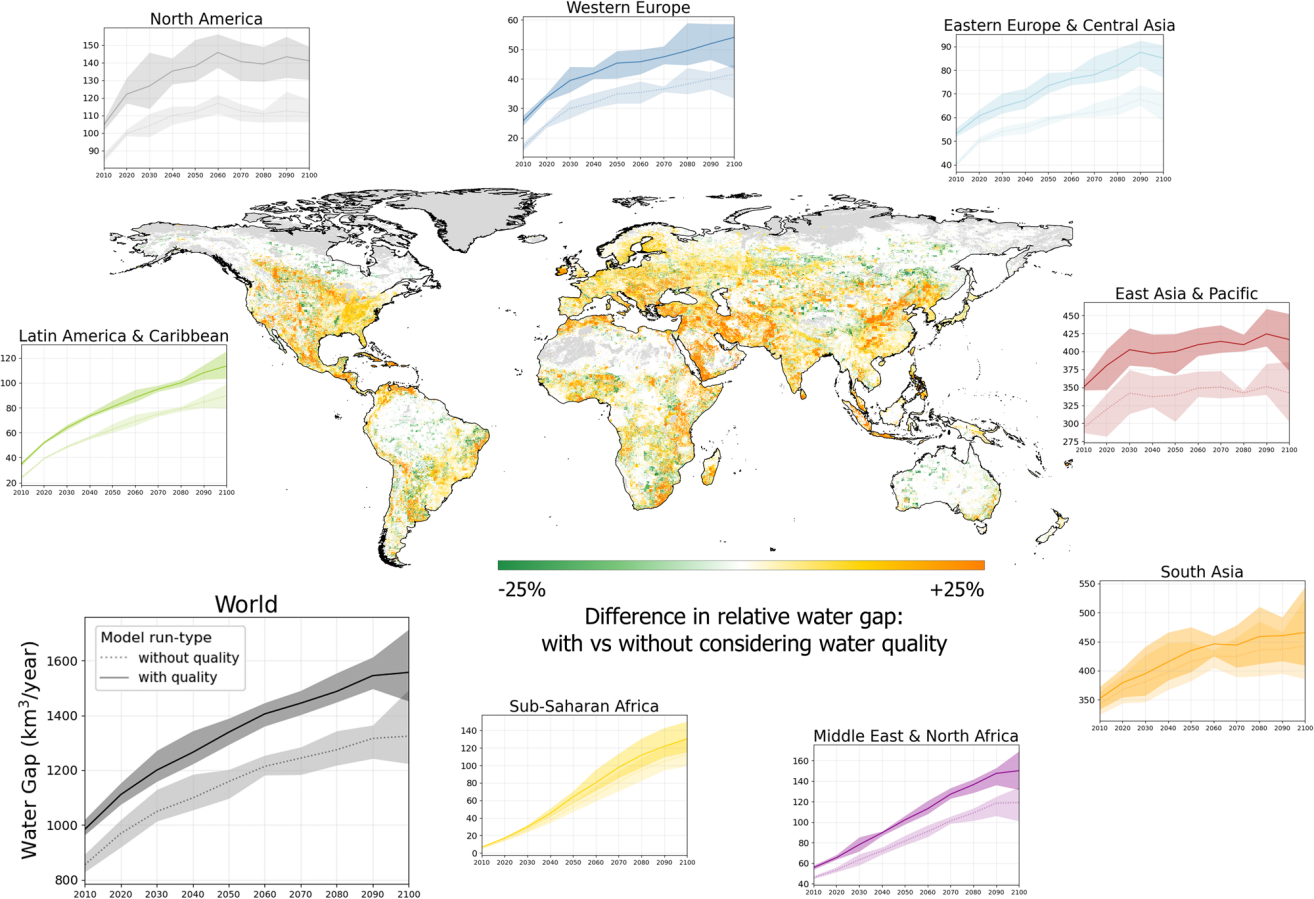
Impacts of considering sectoral water quality requirements in projections of future relative water gap. Central map displays the difference in the relative total clean water gap (i.e., relative water gap from all sectors) minus the relative total water gap, for 2081–2100. Orange coloured locations (grid-cells) imply a relative water gap increase when considering water quality requirements, while green coloured locations imply the contrary. Surrounding time-series plots display the comparison over time of the volumetric total (clean) water gap per geographic region for the ensemble mean (middle line) and the range of variation (i.e., minimum and maximum values) of their corresponding simulations using the bias-corrected output of five GCMs (coloured shade) for the global change scenario SSP3-RCP7.0 for 2005–2100. Solid middle line and dark shadings correspond to a simulation considering water quality requirements (i.e., clean water gap), while the dotted middle line and light shadings represent the simulation that disregards them (i.e., water gap).

Further analysis demonstrates that accounting for sectoral water quality requirements leads to a larger relative clean water gap when compared to simulations that disregard water quality requirements for 2081-2100 in several regions (Fig. 6, orange areas). This is also true for the average over most geographic regions (Supplementary Fig. 6), where the Middle East & North Africa (average difference in relative water gaps of 9% over the 21^st^ century) and Latin America & Caribbean (7%) regions are most impacted when accounting for water quality requirements. Increasing clean water gaps experienced in regions like North America, Europe and Central Asia are largely driven by high manufacturing and thermoelectric water demands that cannot be satisfied due to higher water temperatures, constraining cooling water use purposes. South Asia exhibits the lowest relative water gap difference (average difference in relative water gaps of 2%) by including water quality requirements (Supplementary Fig. 6).

Additionally, results display areas where future water gap decreases when sectoral water quality requirements are considered (Fig. 6, green areas). This results from the adaptative water allocation process in QUAlloc v1.1 (Supplementary Text 2) and the effect of the cross sectoral competition for water of suitable quality. That is, if the water quality requirements for a single sector are unmet due to unsuitable quality conditions, remaining sectors with less stringent water quality requirements in the same location (grid-cell) and surrounding areas can use this water and better meet their demands; hence, decreasing their water gap.

**Fig. 7:**
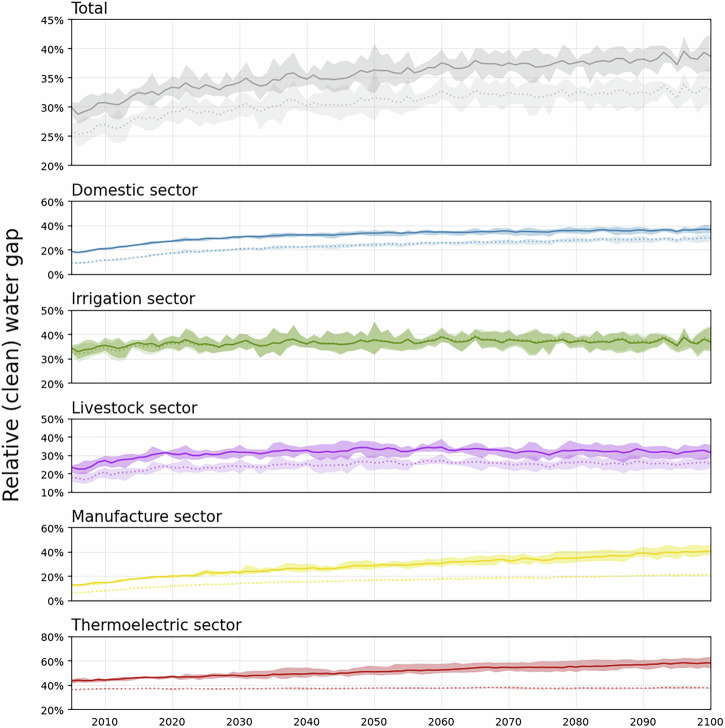
Relative water gap versus relative clean water gap over the period 2005–2100, total and per sector (i.e., domestic, irrigation, livestock, manufacturing, and thermoelectric). Displayed results correspond to the ensemble mean (middle line) and the range of variation (i.e., minimum and maximum values) of their corresponding simulations using the bias-corrected output of five GCMs (coloured shade) for the global change scenario SSP3-RCP7.0. Solid middle line corresponds with the relative clean water gaps, while the dotted middle line represents the relative water gap. Results are aggregated at the yearly time scale.

Our results show that the water gap is projected to increase for all water use sectors under climate change, with or without considering water quality (Fig. 7). Moreover, comparing the long-term development of the water gap shows that the clean water gaps (i.e., when water quality is considered) are consistently larger. The manufacturing and thermoelectric sectors stand out as the most affected sectors when water quality requirements are considered. Such sectors present average relative water gap differences of 12% and 14%, respectively (Fig. 7), mainly affected by rising surface water temperatures, constraining cooling water use. Converse behaviour is shown by the domestic and irrigation sectors, which benefit from the cross-sectoral competition for unused water by the manufacturing and thermoelectric sectors. Here, some locations (grid-cells) with higher differences in relative water gap for the manufacturing sector led to smaller differences for the domestic and, to a lesser extent, the irrigation sector (e.g., Pacific China, Southern Africa or Northern Latin America) (Supplementary Fig. 7).

### Population exposed to water gap

**Fig. 8:**
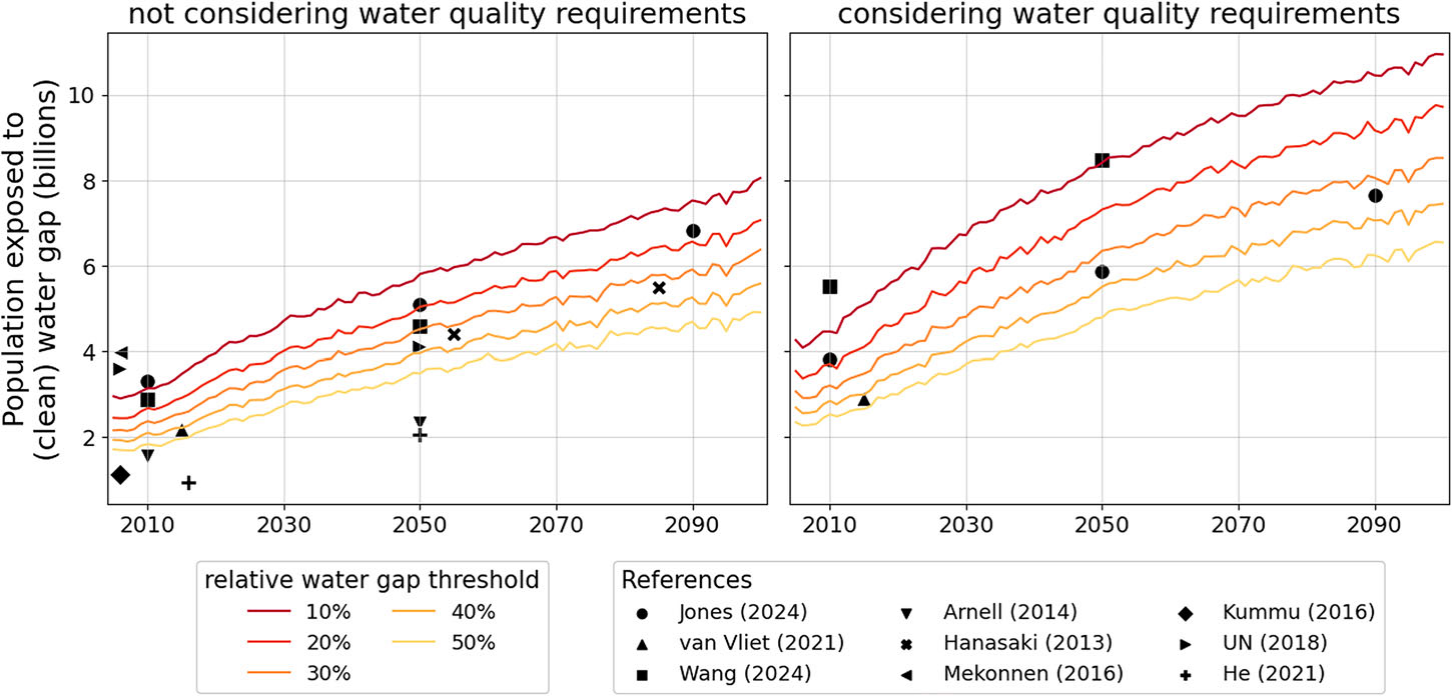
Comparison of population exposed to total (clean) water gap (in billions) under different relative water gap thresholds (i.e. from 10% to 50%) for at least one month per year. Results of population exposed are presented for this study considering different relative water gap thresholds (lines) and compared to estimates of previous studies (black symbols).

Figure 8 shows the population exposed at least one month per year to different values of the relative (clean) water gap. Across all threshold levels, our results show a consistent increase in the global population affected by (clean) water gaps over the 21^st^ century, regardless of whether the sectoral water quality requirements are considered. However, when water quality constraints are included, the rise in population exposed to clean water gaps is more rapid.

The range of global population exposed to total (clean) water gap notably varies depending on the relative water gap threshold used, from 2.4 (50% water gap threshold) to 4.3 billion people (10% threshold) for 2005 when accounting for water quality requirements. Nevertheless, distinct increases in population exposed to water gap are projected over time, particularly for the projections that consider water quality requirements.

A relative water gap threshold of 30% (middle estimate) showcases magnitudes of population exposed to severe water gap plausibly comparable to other relevant research on water scarcity^1,8,13,27^. Our results suggest that neglect of water quality status and sectoral water quality requirements could lead to an underestimation of approximately 38% of the population (1.7 billion people) exposed to a severe clean water gap. Overall, the range of population exposed to the present and future clean water gap (i.e., considering water quality requirements) for the different thresholds are in line with previous water scarcity studies that explicitly accounted for water quality^1,13,17^.

## Discussion

Previous studies evaluated past and future water scarcity mainly as a result of changing physical water availability and demand^6,8,15,28,29^. While recent studies have explicitly incorporated water quality considerations into their analyses^1,13,17^, these assessments were presented with water scarcity aggregated across all sectors. Our study is the first to disentangle the cross-sectoral dynamics that arise when sector-specific water quality requirements are explicitly considered globally under the impact of future climate and socioeconomic change, and to quantify the resulting sector-specific water gaps.

Our results indicate that the deterioration of water quality will exacerbate future water gaps globally, either as a standalone driver (24% of the global inhabited areas) or in combination with insufficient water availability (26%). This is reflected by a substantial increase in the global population experiencing water gaps when water quality is considered. This underpins previous research that addresses future water scarcity in terms of water quality^13,17^. Comparing our new estimates with other relevant water scarcity research (Fig. 8), we found that the global population exposed to severe clean water gap (for the middle range 30% threshold) are consistent with the clean water scarcity estimates reported by Jones et al. (2024)^13^: around 65% by the end-of-century ( ~ 8.1 billion). Similarly, when sector-specific water quality requirements are disregarded, our estimates of the global population exposed to severe water gap by mid- (4.7 billion) and end-of-century (5.7 billion) slightly exceed those reported by Hanasaki et al. (2013)^8^: 4.3–4.5 and 5.3–5.5 billion, respectively, and by the United Nations (2018)^27^: 4.1 billion by 2050. It is important to emphasize the fundamental differences among water scarcity studies (Fig. 8), which partly arise from variations in population datasets used (e.g. global change scenarios applied) as well as the diversity of water scarcity indicators employed (e.g., water shortage, blue water footprint, water stress, water gap) and underlying assumptions (e.g., considering only urban population, or treating surface water as the sole water source).

Our analysis is based on a global change scenario that combines the Shared Socioeconomic Pathway 3 with the Representative Concentration Pathway 7.0. Under this scenario, strong population growth is projected, intensifying anthropogenic pressures on water resources quantity and quality. Such conditions may therefore position our clean water gaps estimates on the upper tier when assessed under climate change scenarios with lower population growth (e.g., SSP1, SSP5), reduced sectoral water demands, and consequently lower pressures on water quantity^30^ and quality^12^. To address climatic uncertainty, we used the ensemble mean of five Global Climate Models; however, relying on a single global change scenario limits our ability to capture a broader range of human and environmental responses. While our results are consistent with previous water scarcity assessments, their robustness could be enhanced by evaluating additional global change scenarios to better represent projection uncertainty and is an opportunity for future research.

A sensitivity analysis of the clean water gaps to the climate projections reveals that higher uncertainties are observed at the end of the century (Supplementary Fig. 8). These uncertainties occur in regions with distinct and contrasting climates: humid areas like the Amazon and central Africa, as well as chronically water scarce regions like central Australia, southern Sahara and the Horn of Africa―most of which are sparsely populated. These findings suggest that, although increasing levels of clean water gaps are simulated in the future (Fig. 2), these results remain uncertain in regions with pronounced climatic characteristics (either humid or dry) and scarcely populated.

The manufacturing and thermoelectric sectors are projected to be the most affected when water quality requirements are considered (relative water gap increases approximately 20% for each sector), primarily due to rising water temperatures. In regions where both sectors demand large volumes of water (e.g., Western Europe or North America), inclusion of sector-specific water quality requirements reduces cross-sectoral competition for water, enabling other users (e.g., domestic or irrigation sectors) to partially reduce their water gaps, as far as the water meets their quality requirements. Although our current model configuration assumes equal prioritization across sectors for water use, these may vary between countries due to specific water management policies. These priorities are predominantly policy-driven and location-specific, often granting preferential access to domestic and irrigation sectors^31,32^, which may lead to an overestimation of their (clean) water gaps in our analysis. Nevertheless, this equal sectoral prioritization assumption allows to assess a competition for clean water availability determined by the conditions of the system (i.e., water quality status).

Our results highlight Africa and the Middle East as the most vulnerable regions in terms of future clean water gaps. By the end-of-century, Sub-Saharan Africa is projected to experience severe water challenges primarily due to the sharp increases in domestic water demands (1225%). This increase additionally contributes towards the deterioration of water quality, leading to an increase in total clean water gap of nearly 2000%. Existing literature shows fewer uniform trends of future water scarcity in Sub-Saharan Africa but generally agrees on an increase in population impacted by a shortage of water, more exposure to water-borne diseases and a declining food production^33–35^. It is additionally highlighted that water scarcity problems in Sub-Saharan Africa are also related to limited accessibility to clean water rather than its unavailability^36^.

Global scale assessments are inherently subject to modelling uncertainties, which stem from user-preference parametrizations and the simplification of the physical processes in the models employed (i.e., QUAlloc v1.1, PCR-GLOBWB 2 and DynQual v1.0). Additionally, these models are currently integrated in a unidirectional manner, which limits their ability to capture certain water use-water quality feedbacks, for instance, changes in sector-specific water abstractions arising from the dynamic allocation process in QUAlloc v1.1 may, in turn, influence pollutant emission levels. Currently, a fully coupled framework is under development, integrating the hydrological- and water quality-related dynamics to the water allocation process.

Environmental flow requirements―although incorporated into previous water scarcity assessments^1,37,38^―are not explicitly included in our analysis, largely due to the complexity of defining them. Environmental flows encompass both quantity components (i.e., discharge)^39^ and water quality dimensions essential to maintain ecosystem health. The quantity-driven (i.e., discharge) nature of the environmental flow assessment highlights opportunities to explicitly incorporate ecological water quality requirements. Advancing this would be a valuable direction for future research, though it will require detailed ecological analyses.

The absence of reliable projections for future water-related information at a global scale remains a critical challenge, necessitating broad assumptions that may introduce potential inaccuracies. For instance, the absence of spatially explicit and exhaustive information on future power plants―including their locations and expected generation capacities―limits the accuracy of thermoelectric water demand projections. Likewise, the assumption that irrigation water withdrawals are equivalent to irrigation water demands (see *Modelling assumptions under a global change scenario* section) has limited impact on estimating actual water withdrawals from QUAlloc v1.1. However, this assumption may lead to an underestimation of the irrigation water gap, particularly in water scarce regions (i.e., the Middle East & North Africa). Moreover, other relevant water-related components, like globally consistent data on transboundary water transfers and spatially explicit projections of wastewater treatment capacity and desalination plants are currently unavailable. While these constraints reflect limited-adaptation conditions and may lead to an overestimation of future clean water gaps, particularly for the domestic, manufacturing and irrigation sectors, addressing these gaps represents an important opportunity for future research towards improved clean water gap projections.

The lack of comprehensive and global-scale groundwater quality projections needs the assumption that all abstracted groundwater meets sector-specific quality standards. This results in conservative estimates and the potential underestimation of the clean water gap in regions where groundwater pollution is already widespread. For instance, several regions identified as experiencing clean water gap challenges―like the central United States, northern India, and central to northern China―are also affected by groundwater salinity pollution^40,41^, which further exacerbates their clean water gap conditions (Supplementary Fig. 9). Similarly, regions in Mediterranean countries such as Spain, Morocco, and Tunisia, as well as northern India, may experience more severe clean water gaps due to high nitrate concentrations in groundwater^42^; in northern India, this problem is further compound by elevated arsenic levels in groundwater^43^. It should also be acknowledged that sectoral water quality standards can vary across regions. However, our use of globally applicable thresholds allows for consistently comparing the impacts of water quality on sectoral water use across various world regions. Similarly, limiting the analysis to four water quality constituents (i.e., water temperature, salinity, organic and pathogen pollution) excludes a broader range of harmful substances to human health and relevant for instance for domestic and irrigation water use (e.g., heavy metals, PFAS, pesticides). While this may lead to an underestimation of the clean water gap, the four constituents evaluated are essential and widely measured indicators of water quality that constrain sectoral water use and determine ecosystem health^44–47^.

Despite these limitations, our study results in a first estimate of future sector-specific clean water gaps, considering water quality requirements across sectors, employing a state-of-the-art hydrological, water quality and water allocation modelling framework applied globally and at a high spatial-temporal resolution. Our study suggests that the combined effect of insufficient water quantity and unsuitable water quality is the most influential driver of the clean water gap under future global change, with conditions expected to worsen in regions with limited water availability, such as Africa and the Middle East, which are projected to face severe clean water gap by the end-of-century. Our findings emphasise that inclusion of water quality requirements yields more alarming projections of increasing sector-specific clean water gaps, reinforcing the urgency of integrated water quality and quantity management strategies.

## Methods

### A modelling framework for sectoral water gap assessment

We employed a novel globally applicable modelling framework that accounts for the dynamics of the main water gap drivers (i.e., low water availability relative to demand and poor water quality), enabling a comprehensive evaluation of sector-specific water gaps. Currently, our framework links in a one-directional way the global hydrological and water resources model PCR-GLOBWB 2^19^ with the global water quality model DynQual v1.0^20^, to the sectoral water use and allocation model QUAlloc v1.0^21^ (Supplementary Text 1). This latter model has been further developed to improve the representation of groundwater extractions and desalination water use, leading to QUAlloc v1.1 (henceforth QUAlloc) (Supplementary Text 2). In our modelling framework, physical water availability per resource and water quality—available from simulations with PCR-GLOBWB 2 and DynQual v1.0 performed at 5 arcmin spatial resolution and at a daily time-step—are passed to QUAlloc, prescribed at a monthly-scale resolution. Further explanation on the coupling and processing steps is detailed in Supplementary Text 1 and Supplementary Fig. 1.

The QUAlloc model assesses water use and allocation for the five major water use sectors (i.e., domestic, irrigation, livestock, manufacturing, and thermoelectric), identifying where and how water is withdrawn and allocated across predefined water allocation zones. To this end, QUAlloc explicitly considers both water availability and water quality requirements, allowing broad assessment of sectoral water use responses to global changes (e.g., increase in water demands). QUAlloc considers surface water, renewable and non-renewable groundwater, and desalinated water as potential water sources to meet sectoral water demands (Supplementary Text 2). Available reusable treated wastewater^48^ is subsequently utilized to mitigate remaining outstanding water demands (Supplementary Fig. 1).

Estimates of available water from renewable sources (i.e., surface water and groundwater), like discharge, total runoff or groundwater storage, are simulated by the global hydrological model PCR-GLOBWB 2^19^ from a setup that assumes no human interference to ensure estimates of full water availability (Supplementary Text 1). Simultaneously, QUAlloc considers estimates of water temperature (Tw) and concentrations of biochemical oxygen demand (BOD) as indicator for organic pollution, total dissolved solids (TDS) to represent salinity and faecal coliform bacteria (FC) as coarse indicator for pathogen pollution in surface water bodies. This water quality constituents are obtained from a DynQual v1.0^20^ simulation coupled to PCR-GLOBWB 2^19^ that accounts for human intervention (Supplementary Text 1).

In combination, the water quantity and quality status were used to determine the clean water availability per sector, based on volumetric demands and sector-specific water quality requirements (Supplementary Table 1). These water quality requirements are presented for the five main water use sectors considered (i.e. domestic, irrigation, livestock, manufacturing, and thermoelectric) and water quality constituents represented in DynQual v1.0 (i.e., water temperature, biochemical oxygen demand, total dissolved solids, faecal coliform bacteria). If surface water quality does not meet the required water quality standards, groundwater is opted for as it is assumed of being of sufficient quality, resulting in a conservative estimate of the calculated water gaps. QUAlloc operates in two stages: *long-term allocation* estimates based on multi-annual water quantity and quality, and *instantaneous allocation* that matches current availability and quality to sectoral water demands. Detailed information on the water allocation based on water availability and quality is presented in Supplementary Text 2 and Supplementary Fig. 2. In addition, QUAlloc proved a strong performance to simulate historical sectoral water withdrawals (1980–2019), showing a high level of agreement between simulated surface water withdrawals from the modelling framework and reported data from AQUASTAT^49^ across different geographic regions^21^.

### Modelling assumptions under a global change scenario

We applied our modelling framework to quantify future sector-specific water gaps under a global change scenario that combines the Shared Socioeconomic Pathway 3 (SSP3) with the Representative Concentration Pathway 7.0 (RCP7.0), aiming to derive estimates of sectoral clean water gap under a scenario with increased human pressures on water resources. To address uncertainties in climate projections, simulations were performed using bias-corrected output of five Global Climate Models (GCMs): gfdl-esm4, ipsl-cm6a-lr, mpi-esm1-2-hr, mri-esm2-0, ukesm1-0-ll selected within the ISIMIP 3b protocol^24^ from the complete CMIP6 ensemble. These GCMs provide the climate variables required by our modelling framework, including total precipitation, daily mean temperature, near-surface relative humidity, surface air pressure, short-wave radiation, and near-surface wind speed, at daily resolution for the period 2000–2100 under the global change scenario SSP3-RCP7.0.

Future sectoral water demand data were pre-processed considering sector-specific environmental and socioeconomic characteristics aligned with the global change scenario SSP3-RCP7.0^26,50^. For domestic, livestock and manufacturing sectors, drivers including population growth and economic development (i.e., Gross Domestic Product) were used to estimate their projected water demands under the global change scenario SSP3-RCP7.0. For the thermoelectric sector, we used the water withdrawal data reported by Lohrmann et al. (2019)^51^ for the reference period (i.e., year 2015) from power plants that rely on freshwater for cooling purposes. In line with scenario SSP3-RCP7.0, such thermoelectric water demands are assumed to remain constant into the future^50^. Irrigation water requirements are dynamically simulated by PCR-GLOBWB 2 at a daily time-step based on hydro-climatic variables like soil moisture in the root zone, crop evapotranspiration and irrigation type^9,19^. For our analysis, we assume the irrigation water demands equivalent to the ensemble mean of the irrigation water withdrawals simulated by PCR-GLOBWB 2 for the five bias-corrected GCMs under the scenario SSP3-RCP7.0, aggregated to a monthly-scale. Similarly, input datasets of water availability and water quality constituents from PCR-GLOBWB 2 and DynQual v1.0, respectively, are obtained from simulations performed at a daily time-step for the period 2005–2100 using ISIMIP 3b bias-corrected data^24^, and aggregated to the monthly-scale (Supplementary Fig. 1). Projections of past and future emission loads of water quality constituents are based on Jones et al. (2024)^13^, which are driven by hydrological projections and sector-specific return flows from PCR-GLOBWB 2. Input Tw, BOD, TDS and FC data excluded values in locations where monthly average discharge was below 0.1 m^3^/s given that uncertainties in absolute water availability strongly affect resulting surface water concentrations^13^. While QUAlloc allows water use from these locations, this has a minimal impact on the allocation results due to the very limited water availability in these areas. Supplementary Table 2 contains the details of the input datasets used to simulate the water allocation and the water gaps and clean water gaps.

Accordingly, simulations of water withdrawal and allocation using QUAlloc were performed at a monthly temporal resolution for the period 2005–2100, at the global-scale and a spatial resolution of 5 arcmin ( ~ 10 km). These simulations employed the bias-corrected output of the five GCMs under the global change scenario SSP3-RCP7.0, while accounting for sector-specific water quality requirements (Supplementary Table 1). To assess the impacts of future global change on water gaps independent of water quality, we also simulated a baseline scenario where sectoral water quality requirements are disregarded, thereby facilitating evaluation of the role of water quality through direct comparisons of “*water gaps*” and “*clean water gaps*”. For the water allocation process, all sectors were assumed to have equal priority, allowing an assessment of sectoral competition that is fully driven by the water quantity and quality conditions of the system. Our modelling framework took two months to provide all output for calculating the (clean) water gap on the Dutch National Supercomputer (Snellius).

### Sectoral water gap estimation

Our analysis builds upon the concept of “water gaps”, i.e., the amount of outstanding water demands after water is supplied from renewable (i.e., surface water and groundwater) and unconventional water sources (i.e., desalinated water and reused wastewater)^14^, as an indicator of water scarcity. We selected this indicator to evaluate the impact of water quality requirements on the clean water gap for the main water use sectors and to contrast it with the water gap driven solely by insufficient water availability. Water supplied from non-renewable sources (e.g., groundwater that is not replenished over human time scale) is disregarded from the analysis as it is considered as a permanent loss of water storage^19,52^. Therefore, our (clean) water gap estimates represent more accurately the water used sustainably to prevent water scarcity. The sector-specific (clean) water gap is determined by the following general equation:

1 $${{{WG}}_{{sector}}}_{t}={{D}_{{sector}}}_{t}-{{\left({A}_{{sw}}+{A}_{{gw}}+{A}_{{dw}}+{A}_{{rww}}\right)}_{{sector}}}_{t}$$

Where: *WG* stands for water gap, *D* for water demands, *A* for water allocated from different water sources: *sw* for surface water, *gw* for renewable groundwater, *dw* for desalinated water, *rww* for reused wastewater; and *t* for the time-step under analysis (i.e., monthly timeseries).

Water allocated to the sectors from surface water (*A*_*sw*_), renewable groundwater (*A*_*gw*_) and desalinated water (*A*_*dw*_) was obtained from the simulations performed with QUAlloc ―within the Water Scarcity Modelling Framework― which captures the cross-sectoral dynamics from competition for limited clean water resources. We assumed that only domestic and manufacturing sectors use desalinated water (*dw*) to meet their clean water demands as ~95% of the global production of desalinated water is used by these two sectors^53,54^. Likewise, we assume that reuse of treated wastewater (*rww*) is for irrigation purposes only, since agriculture is the main user of this water source in multiple regions across the world^55–57^. In addition, projections show an increased dependence on treated wastewater in the future^48,58,59^. Although treated wastewater is also utilized by the industry sector (i.e., manufacturing and thermoelectric), it is primarily for cooling purposes within closed-loop systems that prevent its re-entry into the broader water system^60^. Additionally, to prevent overestimation of the (clean) water gaps, locations (grid-cells) with water demands from only a single sector totalling less than 20 m^3^/year (~0.05 m^3^/day) were excluded.

Calculations of (clean) water gap were performed at a monthly level and per sector for the model simulations considering (i.e., clean water gap) and not considering (i.e., water gap) water quality requirements. Also, (clean) water gaps at the annual-scale are derived by aggregating monthly-scale (clean) water gaps, while total (clean) water gaps are calculated as the sum of (clean) water gaps across all sectors. For intercomparison among the water use sectors or geopolitical and water management units (e.g., countries or river basins), we consider the (clean) water gap relative to its correspondent water demand.

### Cross-sectoral water gap analysis

We identify three types of water gap drivers: water quantity-based (i.e., insufficient water availability to meet sectoral water quantity demands), water quality-based (i.e., exceedance of water quality thresholds for sectoral use), and the combination of them (i.e., that both water quantity and quality issues drive the water gap). Water quality driven water gaps are ascribed if only clean water gaps are obtained. If estimates of water gaps and clean water gaps are identical, these water gaps are ascribed as water quantity driven as there is no additional impact when water quality requirements are considered. Finally, if water gaps are obtained across both simulations, yet are larger due to the inclusion of sectoral water quality requirements (i.e., clean water gaps), then a combined effect of both drivers is ascribed.

Predominant water gap drivers in both the future and reference periods were evaluated by identifying the most recurrent driver (mode) across the period of analysis, based on the annually identified water gap drivers. To assess the impacts of sectoral water quality requirements on clean water gap over time we evaluated the change in relative clean water gap in the future (2081–2100) compared to the reference period (2005–2020). To evaluate regional differences, clean water gaps are grouped by geographic region^61^ and presented as annual means every 20 years. Similarly, to identify seasonal patterns over the 21^st^ century, we present the multiannual monthly means of the clean water gaps every 20 years grouped per geographic region. To assess the effect of accounting for water quality we additionally compared our estimates of the water gap with those of the clean water gap.

We quantify the population exposed to water gap for at least one month during a year using different critical limits (hereafter, relative water gap thresholds), beyond which population experience severe (clean) water shortages. Our results are compared to estimates of population affected by (severe) water scarcity obtained from previous relevant research^1,6,8,13,15,17,28,29^. For consistency, previous estimates are taken for the global change scenario SSP3-RCP7.0 when available, and otherwise by averaging the results of the scenarios used in the previous estimates. Supplementary Table 3 shows the difference in model, scenarios, forcings and criterion among the water scarcity assessments used.

## Supplementary information

Supplementary Information

## Data Availability

Simulated global water allocated and withdrawn for the domestic, irrigation, livestock, manufacturing and thermoelectric sectors at a 5 arcmin (~10 km) resolution for the period 2005-2100 for the global change scenario SSP3-RCP7.0 are openly available at 10.24416/UU01-18V6A3^62^. Datasets are available (1) at the monthly scale for the five GCMs and their ensemble mean, and (2) at the yearly scale for the ensemble mean of the five GCMs.

## Code availability

The PCR-GLOBWB 2 global hydrological model (https://github.com/UU-Hydro/PCR-GLOBWB_model), the DynQual v1.0 surface water quality model (https://github.com/SustainableWaterSystems/DynQual) and the QUAlloc v1.1 water use and allocation model (https://github.com/SustainableWaterSystems/QUAlloc) are available open-access through GitHub. Input datasets used can be found in Supplementary Table 2.
