# Supplementary material for: Sector-specific clean water scarcity and competition under global change
Source: NPJ Clean Water. 2026 Aug 10;9(1):58. doi: 10.1038/s41545-026-00608-0 (PMC13457087; doi:10.1038/s41545-026-00608-0)
Supplement: Supplementary file 1 [file 41545_2026_608_MOESM1_ESM.pdf]

Supplementary Information

Sector-specific clean water scarcity and competition under global change

Gabriel A. Cárdenas Belleza[1,*], Rens L.P.H. van Beek[1], Edward R. Jones[1], Marc F.P. Bierkens[1], Michelle T.H. van Vliet[1]

[1] Department of Physical Geography, Utrecht University, The Netherlands

* Correspondence to: g.a.cardenasbelleza@uu.nl

Contents

Supplementary Text 1 Water Scarcity Modelling Framework

Supplementary Text 2 QUAlloc – Sectoral water use and allocation modelling workflow

Supplementary Text 3 QUAlloc v1.1 model – water allocation processing

Supplementary Fig. 1 Water scarcity modelling framework: interaction between QUAlloc v1.1, PCR-GLOBWB 2, and DynQual v1.0.

Supplementary Fig. 2 Workflow of the water use and allocation model QUAlloc v1.1, per time-step.

Supplementary Fig. 3 Recent past water gap drivers: water quantity, water quality and their combined effect (2005-2020).

Supplementary Fig. 4 Change in the percentage of inhabited areas affected by water gap drivers: water quantity, water quality and their combined effect (past versus future).

Supplementary Fig. 5 Relative clean water gap over the period 2005-2100, per geographic region and sector: domestic, irrigation, livestock, manufacturing, and thermoelectric.

Supplementary Fig. 6 Relative (clean) water gap per geographic region over the period 2005-2100.

Supplementary Fig. 7 Difference in sectoral relative (clean) water gap: considering versus not sectoral water quality requirements, for the period 2081-2100.

Supplementary Fig. 8 Sensitivity analysis of the influence of Global Climate Models on projected (clean) water gaps.

Supplementary Fig. 9 Areas affected by groundwater salinity pollution compared with relative clean water gaps over the 21st century.

Supplementary Table 1 Water quality requirements per water use sector and water quality constituent.

Supplementary Table 2 Input datasets used in QUAlloc v1.1 for analysis of future sectoral water use responses when considering water quality requirements under the global change scenario SSP3 RCP7.0.

Supplementary Table 3 Comparison of water scarcity assessment: inputs, models, criteria.

Supplementary Text 1. Water Scarcity Modelling Framework

The Water Scarcity Modelling Framework is a globally applicable tool for estimating sector-specific water gaps while considering the dynamics of the main water gap drivers: reduced water availability relative to sectoral demands; and unsuitable water quality for a given sectoral use. The framework simulates sectoral water withdrawals and allocations by comparing the water demands with the water availability, its quality status, and sector-specific quality requirements, thereby enabling the quantification of (clean) water gaps. Currently, the modelling framework links the global hydrological and water resources model PCR-GLOBWB 2[1] and the global surface water quality model DynQual v1.0[2] to the sectoral water use and allocation model QUAlloc v1.0[3] in a one-directional coupling.

Supplementary Fig. 1 illustrates the model coupling and the processing steps (circled numbers) used to quantify (clean) water gaps. Steps 1 and 2 were performed prior to the simulations with QUAlloc (steps 3a-3c), so that the final model results are used in step 4.

(1) PCR-GLOBWB 2: Water availability simulation

Surface water (e.g., channel storage, discharge, total runoff), groundwater (e.g., storage, recharge, base flow), and desalinated water availability data were simulated with PCR-GLOBWB 2. To ensure estimates of full water availability from these water sources, the simulations were performed under a "no human interference" setup (non-water demands). Simulations were performed at 5 arcmin spatial resolution and output was reported with a daily time-step. Climate forcing used consisted of bias-corrected outputs from five Global Climate Models (GCMs) —gfdl-esm4, ipsl-cm6a-lr, mpi-esm1-2-hr, mri-esm2-0, ukesm1-0-ll— from the ISIMIP 3b protocol[4] and corresponding to the global change scenario that combines the Shared Socioeconomic Pathway 3 (SSP3) with the Representative Concentration Pathway 7.0 (RCP7.0). The climate variables included total precipitation, daily mean temperature, near-surface relative humidity, surface air pressure, short-wave radiation, and near-surface wind speed, provided at daily resolution for the period 2005-2100.

(2) DynQual v1.0: Surface water quality simulation

Estimates of surface water temperature (Tw) and concentrations of biochemical oxygen demand (BOD) as an indicator for organic pollution, total dissolved solids (TDS) to represent salinity, and faecal coliform bacteria (FC) as a coarse indicator for pathogen pollution were simulated using DynQual v1.0. To represent water quality conditions under human influence, these simulations were performed fully coupled with PCR-GLOBWB 2 in a setup that accounts for sectoral water return flows in calculating pollutant loadings. As in step (1), simulations were performed at 5 arcmin and daily time-step, using the same climate forcings (i.e., gfdl-esm4, ipsl-cm6a-lr, mpi-esm1-2-hr, mri-esm2-0, ukesm1-0-ll) under the global change scenario SSP3-RCP7.0 for the period 2005-2100. These results were obtained from Jones et al.[5].

(3) QUAlloc v1.1

Sectoral water use and allocation was simulated using the QUAlloc v1.1 model, building on Cárdenas et al.[3]. Simulations in QUAlloc (steps 3a-3c) were performed at 5 arcmin and a monthly time-step for the period 2005-2100, which require prescribed inputs from PCR-GLOBWB 2 (step 1) and DynQual v1.0 (step 2) at each time-step. Consequently, the daily outputs from steps 1 and 2 were aggregated to monthly resolution. Simulations were performed (*i*) considering and (*ii*) not considering water quality requirements.

(3a) Sectoral water demands

Future water demand information for the water use sectors considered (i.e., domestic, irrigation, livestock, manufacturing, and thermoelectric) are read from prescribed datasets. These demands were pre-processed prior to these simulations and aimed at reflecting sector-specific environmental and socioeconomic characteristics consistent with the SSP3-RCP7.0 global change scenario (more details in Methods section in the main text). This sectoral demand data is then transferred to the *Water allocation* module (step 3c).

(3b) Sectoral water quality

The suitability of using water based on its quality conditions is determined for each water source and sector. To this end, sector-specific quality requirements (Supplementary Table 1) are evaluated against previous (long-term) and instantaneous (short-term) water quality conditions. This suitability assessment considers the fours water quality constituents —Tw, BOD, TDS and FC— provided by the DynQual v1.0 dataset (step 2). The resulting suitability information is then passed to the *Water allocation* module (step 3c).

(3c) Water allocation

Water allocation is assessed by identifying where and how water is withdrawn, passing through a long- and a short-term water allocation stages. To do so, water availability information from surface water, groundwater and desalinated water are read from PCR-GLOBWB 2 dataset (step 1), assessed together with water quality suitability (step 3b) and compared against sectoral water demands (step 3a). The water allocation process assumed diverse water management options (e.g., equal water use priority across sectors, water allocation zones, etc.). Main outputs from QUAlloc v1.1 are sectoral water allocations from surface water, groundwater (renewable and non-renewable), and desalinated water. Detailed information on the water allocation process and assumptions in QUAlloc v1.1 are presented in Supplementary Text 2 and Supplementary Fig. 2.

(4) Sectoral water gap estimation

The sector-specific (clean) water gaps (*WG*) at the monthly time-step (*t*) are determined using Equation 1. Here, *D* stands for sectoral water demands (prescribed input data, Supplementary Fig. 1), *A* for water allocated to the sectors from surface water (A_{sw}), renewable groundwater (A_{gw}) and desalinated water (A_{dw}) (model outputs from QUAlloc v1.1). Water allocated from reuse treated wastewater (A_{rww}) is obtained from Jones et al.[6]. We assumed that desalinated water (*dw*) can only be used by domestic and manufacturing sectors, while reuse of treated wastewater (*rww*) is for irrigation purposes only.

$$WG_{sector_t} = D_{sector_t} - \left(A_{sw} + A_{gw} + A_{dw} + A_{rww}\right)_{sector_t} \qquad (Equation\ 1)$$

Supplementary Text 2. QUAlloc – Sectoral water use and allocation modelling workflow

QUAlloc v1.0[3] is a globally applicable model for simulating water use and allocation through dynamically matching sectoral water demand with available resources across major water use sectors, including domestic, irrigation, livestock, manufacturing, and energy (i.e. thermoelectric power). QUAlloc integrates surface and groundwater availability simulated data from the global hydrological model PCR-GLOBWB 2[1] and water quality simulated with the surface water quality model DynQual v1.0[2], to determine the optimal source and location of water provision at a monthly time-step. The following water quality constituents are represented which are relevant for various sectoral uses: temperature (Tw) and concentrations of total dissolved solids (TDS) as indicator for salinity, biochemical oxygen demand (BOD) to represent organic pollution and faecal coliform bacteria (FC) as a coarse indicator for pathogen pollution.

QUAlloc accounts for both long-term trends and short-term variability in water quantity and quality, enabling adaptive allocation strategies to minimize sectoral shortfalls, each of them representing a water allocation processing stage. During the *long-term stage*, potential water withdrawals are estimated per source and sector based on historical conditions (e.g., water availability and quality) and decisions (e.g., actual water withdrawn) from previous years, to represent the expectations of the locations where enough and good quality water is commonly found. During the *short-term* (hereafter, *instantaneous*) *stage*, these long-term expectations are updated using current hydrological and water quality conditions to determine actual withdrawals per sector.

QUAlloc allows one to define the spatial distribution of source-sink water networks as a feature of potential hydraulic infrastructure to set sectoral water use priorities to guide allocation decisions by water management, and the consideration of water use constraints based on sector-specific water quality requirements (Supplementary Table 1). The water allocation process assumes the existence of infrastructure capable of transporting water across a defined spatial extent where water withdrawals are aggregated to satisfy the sectoral demands. Within each allocation zone, sectors place claims on available water resources so that withdrawals can occur at any location, with abstraction only limited by availability or technological capacity (i.e., pumping capacity). Sectoral claims within overlapping allocation zone are aggregated and distributed considering prioritization rules, if defined, by weighting sectoral water demands using the inverse of their priority levels. This approach gives to lower-priority-sectors a proportionally higher potential withdrawal. The allocation process iterates until all demands are satisfied or the available water is depleted. In this study, equal sectoral prioritization was assumed as this allows to assess a competition for clean water availability determined by the conditions of the system (i.e., water quantity and quality status). In the absence of global water management zone data, the default 1°×1° allocation zones from PCR-GLOBWB 2[1] are used, adjusted to respect national borders and major river basin boundaries.

QUAlloc v1.0[3] was further developed to better represent desalinated water use and groundwater abstractions (i.e., regional pumping capacity). Accordingly, QUAlloc v1.1 incorporates desalinated water production as an additional source to supply water demands for the domestic and manufacturing sectors. Information on desalinated water production was obtained from a licensed global database which compiles data from more than 20,000 desalination plants[7]. Desalinated water available is assumed to be equivalent to the desalination capacity from onshore desalination plants, which is aggregated to 5 arcmin spatial resolution and to an annual time scale for the period 1980 to 2018, which are progressively introduced according to their construction year. For global change simulations, due to the absence of reliable projections at a global-scale, values from latest available year (i.e., 2018) are kept constant over time. Additionally, QUAlloc v1.1 incorporates

the effect of the installed pumping capacity from water sources. Such pumping capacity caps the amount of water that can be withdrawn for a specific source due to physical (installation) limitation, standing as a proxy to estimate (i.e.,) groundwater withdrawal capacity from non-renewable source. We use the groundwater pumping capacity information from PCR-GLOBWB 2 which, in turn, is based on the IGRAC Global Groundwater Information System (GGIS) database and the Food Production Units from the IMPACT project[1,8,9]. Surface water use is assumed to be only limited by its availability, therefore not including a surface water pumping capacity. This information is reported at a regional and annual scales, therefore being spatially distributed to 5 arcmin internally, mainly using the estimates of potential renewable groundwater withdrawals.

The workflow of the water allocation process for each time-step in QUAlloc v1.1 is displayed in Supplementary Fig. 2 and described in the following points:

Long-term allocation stage

[1] ***Withdrawal capacity***. The maximum withdrawal capacity of a specific location (grid-cell) per water source uses the pumping capacity of a region to extract water and is spatially distributed based on the potential long-term withdrawals of the source.

[2] ***Long-term availability (and water quality) per source***. The concept of "long-term" refers to the multiannual monthly average values of a specific variable. In this regard, the potential available surface water and groundwater per location (grid-cell) are determined based on the values of long-term discharge, total runoff and groundwater storage. Thus, the long-term water availability refers to renewable water per source. It additionally considers the withdrawal capacity per source (step 1) and a defined sectoral water use prioritization; a standard QUAlloc v1.1 run considers equal priority for all sectors. Similarly, locations (grid-cells) where water is predominantly of suitable quality are determined based on the long-term water quality data (i.e., Tw, BOD, TDS and FC) per source and the sectoral water quality requirements (Supplementary Table 1). If a water source does not specify any water quality data, the grid-cell is assumed to not have any water use suitability problems, which may result in a conservative estimate of the clean water gap.

[3] ***Water demand per sector***. It defines the gross and net water demand of the domestic, irrigation, livestock, manufacturing and thermoelectric sectors, typically read from prescribed datasets. Here, the sectoral prioritization is updated depending on the extension of the allocation zone of a sector. For instance, since the water use for cooling by the thermoelectric sector is typically withdrawn from nearby surface water sources, its sectoral water use priority for surface water is higher compared to other sectors in the same area. The difference between gross and net water demand results into the potential return flow.

[4] ***Actual desalinated water withdrawal per sector***. Desalinated water is treated as the first source used to satisfy the sectoral demands. Water is aggregated over the allocation zones where desalinated water is produced and distributed accordingly to the sectors that are assumed to use desalinated water (i.e., as of now, only domestic and manufacturing sectors). Here, locations (grid-cells) where water is withdrawn and allocated are determined.

[5] ***Long-term potential withdrawals per source & sector***. The locations (grid-cells) where water is assumed to be withdrawn to satisfy the water demands per water source and sector are determined. This process is based on the long-term water availability and its long-term suitability (step 2) and the outstanding sectoral demand after the desalinated water is being supplied (steps 3 and 4). The water is distributed following a predefined prioritization rule (if specified) over the sectoral demands accounting for their

water quality requirements (Supplementary Table 1) until the availability from renewable sources is exhausted (hereafter, potential long-term renewable withdrawals). After, outstanding water demands are matched to the remaining water withdrawal capacity (considering the already used capacity) to satisfy such demands from non-renewable groundwater if possible (hereafter, potential long-term non-renewable withdrawals).

Instantaneous allocation stage

[6] ***Actual surface water withdrawn per sector***. Potential long-term surface water withdrawals per sector (step 5) are paired to the surface water availability and suitability for the current time-step. The current surface water availability is based on a routed instantaneous total runoff and the instantaneous state of the water quality constituents (i.e., Tw, BOD, TDS and FC). Here, location (grid-cell) and amount of the actual (renewable) surface water withdrawal are determined per sector.

[7] ***Updated potential non-renewable groundwater withdrawals per sector***. Unmet potential long-term surface water withdrawals from step 6 are redistributed over the potential long-term non-renewable groundwater withdrawals (step 5) considering the withdrawal capacity (step 1).

[8] ***Actual groundwater withdrawn per sector***. Updated potential long-term groundwater withdrawals per sector (renewable and non-renewable) (step 7) are paired to the groundwater availability for the current time-step. The current groundwater availability is based on the groundwater storage from previous time-step and the instantaneous recharge and baseflow of the current time-step. Due to the absence of reliable and large-scale groundwater quality data, available groundwater is always assumed of suitable quality. Here, location (grid-cell) and amount of the actual renewable and non-renewable groundwater withdrawals are determined per sector.

[9] ***Met demands per supply, source & sector***. Actual renewable and non-renewable surface water and groundwater withdrawals (steps 6 and 8) represent the locations (grid-cells) where suitable water is abstracted from to satisfy the sectoral demands. Thus, the withdrawn water is allocated over the allocation zones to the locations (grid-cells) where water is demanded for each of the sectors. A water gap results from the unmet water demands after the water allocation.

[10] ***Updated long-term availability, quality & potential withdrawals***. The long-term variables are updated at the end of the year by weighting their running average using by the inverse of the number of years (1/N years) that are expected to take the water users to adapt to changes in the system (e.g., deterioration of water quality, development of hydraulic infrastructure, etc.). Standard simulation considers that adaptation takes two years to update the long-term variables.

Details of the calculation steps and equations are included in the Supplementary Text 3.

Supplementary Text 3. QUAlloc v1.1 model – water allocation processing

Acronyms

$$sw = surface\ water \qquad i = pixel\ level$$
$$gw = groundwater \qquad a = allocation\ zone\ level$$
$$dw = desalinated\ water \qquad r = regional\ level$$
$$rn = renewable \qquad lt = long - term$$
$$nr = non - renewable \qquad st = short - term\ (instantaneous)$$
$$x = sectors \qquad t = time - step\ (monthly)$$
$$c = water\ quality\ constituents \qquad \Delta t = days\ in\ month$$

Model initialization

1. read-in time independent variables

$$A = grid\ cell\ area \quad [m^2]$$
$$\omega_{dw/sw/gw_{x_i}} = prioritization \quad [-]$$
$$a_{dw/sw/gw_{x_{i,t}}} = allocation\ zones \quad [-]$$
$$W_{sw/gw_i}^{points} = withdrawal\ points \quad [-]$$
$$C_{x_{c_i}}^{max} = sectoral\ water\ quality\ requirements \quad [*]$$
$$f_{r_{sw_i}} = surface\ water\ fraction \quad [m^2/m^2]$$
$$\omega_t^{lt} = adaptation\ ratio \quad [1/year]$$

2. update sectoral prioritization based on allocation zones extension

$$\omega_{sw/gw_{x_{i,t}}} = \omega_{sw/gw_{x_i}} \times a_{x_{i,t}} \quad [-]$$

3. read-in long-term variables

$$Q_{i,t}^{lt} = long - term\ discharge\ |_{\ current\ month} \quad [m^3/s]$$
$$R_{i,t}^{lt} = long - term\ total\ runoff\ |_{\ current\ month} \quad [m/d]$$
$$s_{i,t}^{lt} = long - term\ groundwater\ storage\ |_{\ current\ month} \quad [m]$$
$$C_{sw/gw_{c_{i,t}}}^{lt} = long - term\ water\ quality\ constituents\ |_{current\ month} \quad [*]$$
$$\widehat{W}_{sw/gw_{i,t}}^{lt} = long - term\ potential\ water\ withdrawal\ |_{current\ month} \quad [m^3/d]$$

4. read-in initial states

$$h_{i,t-1} = channel\ storage\ |_{\ previous\ time-step} \quad [m]$$
$$Q_{rf_{i,t-1}} = return\ flow\ |_{\ previous\ time-step} \quad [m/d]$$

$$s_{i,t-1} = groundwater\ storage\ |_{\ previous\ time-step} \quad [m]$$

Long-term allocation stage

[1] ***Withdrawal capacity***

1. read-in regional pumping capacities for the current year

$$W^{max}_{sw/gw_{r,year}} = regional\ pumping\ capacity \quad [m^3/year]$$

$$reg_{sw/gw_{\ i,year}} = regional\ ids \quad [-]$$

2. calculate the spatial distribution of potential withdrawals for current time-step

$$r_{sw/wg_{i,t}} = \frac{\max\left(\widehat{W}^{lt}_{sw/gw_{i,t}}\right)\Big|_{across\ the\ year_{\ i}}}{\max\left(\widehat{W}^{lt}_{sw/gw_{i,t}}\right)\Big|_{across\ the\ year_{\ r}}} \quad [m^3/m^3]$$

3. calculate withdrawal capacity

$$W^{max}_{sw/gw_{i,t}} = \frac{W^{max}_{sw/gw_{r,year}} \times r_{sw/wg_{i,t}}}{12 \times \Delta t} \quad [m^3/d]$$

[2] ***Long-term availability (and water quality) per source***

1. get long-term potential surface water availability

$$Q^{lt}_{i,t} = Q^{lt}_{i-1,t} + R^{lt}_{i,t} \times \frac{A}{86400} \quad [m^3/s]$$

$$h^{lt}_{i,t} = f_{Manning}\left(Q^{lt}_{i,t},\ h^{lt}_{i,t-1}\right) \quad [m]$$

$$\widehat{V}^{lt}_{sw_{i,t}} = h^{lt}_{i,t} \times length_{channel} \times width_{channel} \quad [m^3/d]$$

2. get long-term potential groundwater availability

$$\widehat{V}^{lt}_{gw_{i,t}} = s^{lt}_{i,t} \times A \quad [m^3/d]$$

3. limit long-term potential availability with withdrawal capacity

$$\widehat{V}^{lt}_{sw/gw_{i,t}} = min\begin{cases} W^{max}_{sw/gw_{i}} \\ \widehat{V}^{lt}_{sw/gw_{i,t}} \end{cases} \quad [m^3/d]$$

4. get long-term potential water quality suitability per sector

$$f^{lt}_{sw/gw_{x_{i,t}}} = \frac{C^{lt}_{sw/gw_{constituent_{i,t}}}}{C^{max}_{x_{constituent_{i}}}}\Bigg|_{[0-1]} \quad [-]$$

[3] ***Water demand per sector***

1. read-in water demands for all sectors for the current time-step

$$\widehat{D}_{x_{\ i,t}} = gross\ water\ demands\ |_{\ current\ month} \quad [m/d]$$

2. update sectoral gross water demand units to volume

$$\widehat{D}_{x_{i,t}} = \widehat{D}_{x_{i,t}} \times A \quad [m^3/d]$$

[4] ***Actual desalinated water withdrawal per sector***

1. get short-term desalinated water availability

$$V_{dw_{i,t}}^{st} = desalinated\ water\ use\ |_{\ current\ month} \quad [m^3/d]$$

2. distributes the desalinated water to the selected sectors (i.e., domestic and manufacture)

$$start\ loop:$$

i. get distributing weights per sector

$$w_{dw_{x\,i,t}} = \frac{1}{\omega_{dw\,x_{i,t}}} \times \frac{\widehat{D}_{x_{a,t}}}{\widehat{D}_{a,t}} \times \frac{V_{dw_{i,t}}}{\bar{V}_{dw_{i,t}}} \quad [-]$$

ii. update desalinated water use (withdrawal) per sector

$$W_{dw\,i,t} \mathrel{+}= V_{dw_{i,t}}^{st} \times w_{dw_{x\,i,t}} \Big|_{\substack{1^{st}\ local \\ 2^{nd}\ zonal}} \quad [m^3/d]$$

$$end\ loop.$$

3. update unmet demands

$$\widehat{D}_{x_{i,t}} = \widehat{D}_{x\,i,t} - W_{dw\,i,t} \quad [m^3/d]$$

[5] ***Long-term potential withdrawals per source & sector***

1. distributes potential renewable water available per source to the sectors; thermoelectric demands can be met only from surface water and local sources

$$start\ loop:$$

i. get distributing weights per sector

$$w_{sw/gw_{x\,i,t}} = \frac{1}{\omega_{sw/gw_{\,x_{i,t}}}} \times \frac{\widehat{D}_{x_{a,t}}}{\widehat{D}_{a,t}} \times \frac{f_{sw/gw_{x_{i,t}}} \times \hat{V}_{sw/gw_{\,i,t}}}{\dfrac{\sum_{i=1}^{a}\left(f_{sw/gw_{x_{i,t}}}^{lt} \times \hat{V}_{sw/gw_{\,i,t}}\right)}{\sum_{i=1}^{a}\left(f_{sw/gw_{x_{i,t}}}^{lt}\right)}} \quad [-]$$

ii. get potential available water per sector

$$\hat{V}'_{sw/gw_{x_{i,t}}} = \hat{V}_{sw/gw_{\,i,t}} \times w_{sw/gw_{x\,i,t}} \times f_{sw/gw_{x_{i,t}}}^{lt} \quad [m^3/d]$$

iii. update long-term potential renewable water withdrawal per sector

$$\widehat{W}_{sw/gw_{rn/nr_{x_{i,t}}}}^{lt} \mathrel{+}= f\left(\hat{V}'_{sw/gw_{x_{i,t}}}, \widehat{D}_{x_{\,i,t}}\right) \Big|_{\substack{1^{st}\ local \\ 2^{nd}\ zonal}} \quad [m^3/d]$$

$$end\ loop.$$

2. get unmet demands

$$\bar{D}_{x_{i,t}} = \widehat{D}_{x_{\,i,t}} - \widehat{W}_{x_{i,t}}^{lt} \quad [m^3/d]$$

3. get potential available groundwater per sector

$$A'_{x_{i,t}} = if\left(W_{gw_i}^{points} > 0, ifthenelse\left(\widehat{W}_{gw_{rn_{x_{i,t}}}}^{lt} > 0\,, \widehat{W}_{gw_{rn_{x_{i,t}}}}^{lt} \times f_{gw_{x_{i,t}}}^{lt}\,, W_{gw_i}^{max} \times f_{gw_{x_{i,t}}}^{lt}\right), 0\right) \quad [m^3/d]$$

4. get long-term potential non-renewable groundwater withdrawals by re-distributing unmet demands

$$\widehat{W}_{gw_{nr_{x_{i,t}}}}^{lt} = \overline{D}_{x_{i,t}} \times \frac{A'_{x_{i,t}}}{A'_{x_{a,t}}} \quad [m^3/d]$$

Instantaneous allocation stage

[6] ***Actual surface water withdrawn per sector***

1. read-in climate and surface water related components for current time-step

$$P = precipitation \mid_{current\ month} \quad [m/d]$$

$$ET_{ref} = reference\ evapotranspiration \mid_{current\ month} \quad [m/d]$$

$$k_c = crop\ factor \mid_{current\ month} \quad [-]$$

$$R_d = direct\ runoff \mid_{current\ month} \quad [m/d]$$

$$R_i = total\ interflow \mid_{current\ month} \quad [m/d]$$

$$R_b = base\ flow \mid_{current\ month} \quad [m/d]$$

2. calculate channel runoff

$$C_{H_{i,t}} = P - ET_{ref} \times k_c \quad [m/d]$$

3. calculate surface water total runoff

$$R_{T_{i,t}} = max\begin{cases} 0 \\ R_b + [\, R_d + R_i \,]_{\substack{over \\ land}} + [\, C_{H_{i,t}} \,]_{\substack{over \\ channel}} + Q_{rf_{t-1}} \end{cases} \quad [m/d]$$

4. calculate suitability of current surface water quality

$$f_{sw_{x_{i,t}}}^{st} = \left.\frac{C_{sw_{constituent_i}}^{st}}{C_{x_{constituent_i}}^{max}}\right|_{[0-1]} \quad [-]$$

5. calculate current potential water available in the channel

$$\hat{V}_{sw_{i,t}}^{st} = \left(h_{i,t-1} \times f_{r_{sw_i}} + R_{T_{i,t}}\right) \times A \quad [m^3/d]$$

6. aggregate potential surface water withdrawals by sector

$$\widehat{W}_{sw_{x_{i,t}}}^{lt} = \widehat{W}_{sw_{rn_{x_{i,t}}}}^{lt} + \widehat{W}_{sw_{nr_{x_{i,t}}}}^{lt} \quad [m^3/d]$$

7. calculate current potential surface water withdrawals per sector

$$\widehat{W}_{sw_{x_{i,t}}}^{st} = \widehat{W}_{sw_{x_{i,t}}}^{lt} \times f_{sw_{x_{i,t}}}^{st} \quad [m^3/d]$$

8. update current potential surface water withdrawals based on availability

$$\widehat{W}^{st}_{sw_{i,t}} = \sum_{x=sector}^{sectors} \widehat{W}^{st}_{sw_{x_{i,t}}} \quad [m^3/d]$$

9. update current potential surface water withdrawals based on availability

$$\widehat{W}^{st}_{sw_{x_{i,t}}} = min \begin{cases} \widehat{W}^{st}_{sw_{x_{i,t}}} \\ \widehat{V}^{st}_{sw_{i,t}} \times f^{st}_{sw_{x_{i,t}}} \times \dfrac{\widehat{W}^{st}_{sw_{x_{i,t}}}}{\widehat{W}^{st}_{sw_{i,t}}} \end{cases} \quad [m^3/d]$$

10. aggregate updated current potential surface water withdrawals by sector

$$\widehat{W}^{st}_{sw_{i,t}} = \sum_{x=sector}^{sectors} \widehat{W}^{st}_{sw_{x_{i,t}}} \quad [m^3/d]$$

11. convert total runoff units from water slice to volume

$$R_{T_{i,t}} = R_{T_{i,t}} \times A \quad [m^3/d]$$

12. routing total runoff and get actual water withdrawal

$$W_{sw_{i,t}} = accuthresholdstate\left(ldd,\ R_{T_{i,t}},\ \widehat{W}^{st}_{sw_{i,t}}\right) \quad [m^3/d]$$

$$Q_{i,t} = accuthresholdflux\left(ldd,\ R_{T_{i,t}},\ \widehat{W}^{st}_{sw_{i,t}}\right) / 86400 \quad [m^3/s]$$

13. update channel storage for current time-step

$$h_{i,t} = f_{Manning}\left(Q_{i,t},\ h_{i,t-1}\right) \ [m]$$

14. re-distribute actual water withdrawals per sector

$$W_{sw_{x_{i,t}}} = W_{sw_{i,t}} \times \frac{\widehat{W}^{st}_{sw_{x_{i,t}}}}{\widehat{W}^{st}_{sw_{i,t}}} \quad [m^3/d]$$

[7] ***Updated potential non-renewable groundwater withdrawals per sector***

1. calculate unmet potential surface water withdrawals per sector

$$\overline{D}_{x_{i,t}} = \widehat{W}^{lt}_{sw_{x_{i,t}}} - W_{sw_{x_{i,t}}} \quad [m^3/d]$$

2. get potential available groundwater per sector

$$A'_{x_{i,t}} = if\left(W^{points}_{gw_i} > 0, ifthenelse\left(\widehat{W}^{lt}_{gw_{rn_{x_{i,t}}}} > 0, \widehat{W}^{lt}_{gw_{rn_{x_{i,t}}}} \times f^{lt}_{gw_{x_{i,t}}}, W^{max}_{gw_i} \times f^{lt}_{gw_{x_{i,t}}}\right), 0\right) \quad [m^3/d]$$

3. re-distribute unmet potential withdrawals to non-renewable groundwater

$$\widehat{W}^{lt}_{gw_{nr_{x_{i,t}}}} += \overline{D}_{x_{i,t}} \times \frac{A'_{x_{i,t}}}{A'_{x_{a,t}}} \quad [m^3/d]$$

4. aggregate potential groundwater withdrawals by supply (and sector)

$$\widehat{W}^{lt}_{gw_{x_{i,t}}} = \widehat{W}^{lt}_{gw_{rn_{x_{i,t}}}} + \widehat{W}^{lt}_{gw_{nr_{x_{i,t}}}} \quad [m^3/d]$$

$$\widehat{W}_{gw_{i,t}}^{lt} = \sum_{x=sector}^{sectors} \widehat{W}_{gw_{x_{i,t}}}^{lt} \quad [m^3/d]$$

[8] ***Actual groundwater withdrawn per sector***

1. read-in groundwater related components for current time-step

$$r_{T_{i,t}} = total\ recharge \quad [m/month]$$

2. calculate daily actual groundwater recharge

$$r_{i,t} = \frac{r_{T_{i,t}}}{\Delta t} - \frac{\widehat{W}_{gw_{i,t}}^{lt}}{A} \quad [m/d]$$

3. calculate base flow from the previous time-step

$$bf_{i,t-1} = base\ flow \mid_{previous\ time-step} = \alpha \times s_{i,t-1} \quad [m/d]$$

4. calculate total base flow at the end of the period

$$for\ day\ in\ month:$$

$$bf_{i,day} = max \begin{cases} 0 \\ bf_{i,day-1} \times e^{-\alpha \times day} + r_{i,day} \times \left(1 - e^{-\alpha \times day}\right) \end{cases} \quad [m/d]$$

$$bf_{T_{i,t}} += bf_{i,day} \quad [m/month]$$

5. calculate suitability of current groundwater quality

$$f_{gw_{x\,i,t}}^{st} = \left. \frac{C_{gw_{constituent_i}}^{st}}{C_{x_{constituent\,i}}^{max}} \right|_{[0-1]} \quad [-]$$

6. update the current potential groundwater storage

$$\hat{s}_{i,t} = s_{i,t-1} + r_{T_{i,t}} - bf_{T_{i,t}} \quad [m/month]$$

7. calculate current potential groundwater available

$$\widehat{V}_{gw_{i,t}}^{st} = max \begin{cases} 0 \\ \hat{s}_{i,t} \times A \end{cases} \quad [m^3]$$

8. calculate current potential groundwater withdrawals per sector and supply

$$\widehat{W}_{gw_{x_{i,t}}}^{st} = \widehat{W}_{gw_{x_{i,t}}}^{lt} \times f_{gw_{x\,i,t}}^{st} \times \Delta t \quad [m^3/month]$$

9. aggregate current potential groundwater withdrawals by supply and by sector

$$\widehat{W}_{gw_{i,t}}^{st} = \sum_{x=sector}^{sectors} \widehat{W}_{gw_{x_{i,t}}}^{st} \quad [m^3/month]$$

10. calculate actual renewable and non-renewable groundwater withdrawals

$$W_{gw_{rn_{i,t}}} = min \begin{cases} \widehat{V}_{gw_{i,t}}^{st} \\ \widehat{W}_{gw_{i,t}}^{st} \end{cases} \quad [m^3/month]$$

$$W_{gw_{nr_{i,t}}} = max \begin{cases} 0 \\ \widehat{W}_{gw_{i,t}}^{st} - W_{gw_{rn_{i,t}}} \end{cases} \quad [m^3/month]$$

11. re-distribute actual renewable and non-renewable water withdrawals per sector

$$W_{gw_{rn/nr_{x_{i,t}}}} = W_{gw_{rn/nr_{i,t}}} \times \frac{\widehat{W}^{st}_{gw_{x_{i,t}}}}{\widehat{W}^{st}_{gw_{i,t}}} \times \frac{1}{\Delta t} \quad [m^3/d]$$

12. update groundwater storage for current time-step

$$s_{i,t} = s_{i,t-1} + r_{T_{i,t}} - bf_{T_{i,t}} - \left(W_{gw_{rn_{i,t}}} + W_{gw_{nr_{i,t}}}\right) \times \frac{\Delta t}{A} \quad [m]$$

[9] ***Met demands per supply, source & sector***

1. allocate actual withdrawals to demands per sector

$$Al_{sw/gw_{rn/nr_{x_{i,t}}}} = \frac{D_{x_i}}{D_{x_a}} \times W_{sw/gw_{rn/nr_{x_{a,t}}}} \Bigg|_{\substack{1^{st}\ local \\ 2^{nd}\ zonal}} \quad [m^3/d]$$

[10] ***Updated long-term availability, quality and potential withdrawals***

1. update long-term variables to calculate availability, quality and potential withdrawals corresponding to same month of next year

$$Q^{lt}_{i,t+12} = \left(\omega^{lt}_t \times Q_{i,t}\right) + \left(\left(1 - \omega^{lt}_t\right) \times Q^{lt}_{i,t}\right) \quad [m^3/s]$$

$$R^{lt}_{i,t+12} = \left(\omega^{lt}_t \times R_{i,t}\right) + \left(\left(1 - \omega^{lt}_t\right) \times R^{lt}_{i,t}\right) \quad [m/d]$$

$$s^{lt}_{i,t+12} = \left(\omega^{lt}_t \times s_{i,t}\right) + \left(\left(1 - \omega^{lt}_t\right) \times s^{lt}_{i,t}\right) \quad [m]$$

$$C^{lt}_{sw/gw_{c_{i,t+12}}} = \left(\omega^{lt}_t \times C^{st}_{sw/gw_{c_{i,t}}}\right) + \left(\left(1 - \omega^{lt}_t\right) \times C^{lt}_{sw/gw_{c_{i,t}}}\right) \quad [*]$$

$$\widehat{W}^{lt}_{sw/gw_{i,t+12}} = \left(\omega^{lt}_t \times \widehat{W}^{st}_{sw/gw_{i,t}}\right) + \left(\left(1 - \omega^{lt}_t\right) \times \widehat{W}^{lt}_{sw/gw_{i,t}}\right) \quad [m^3/d]$$

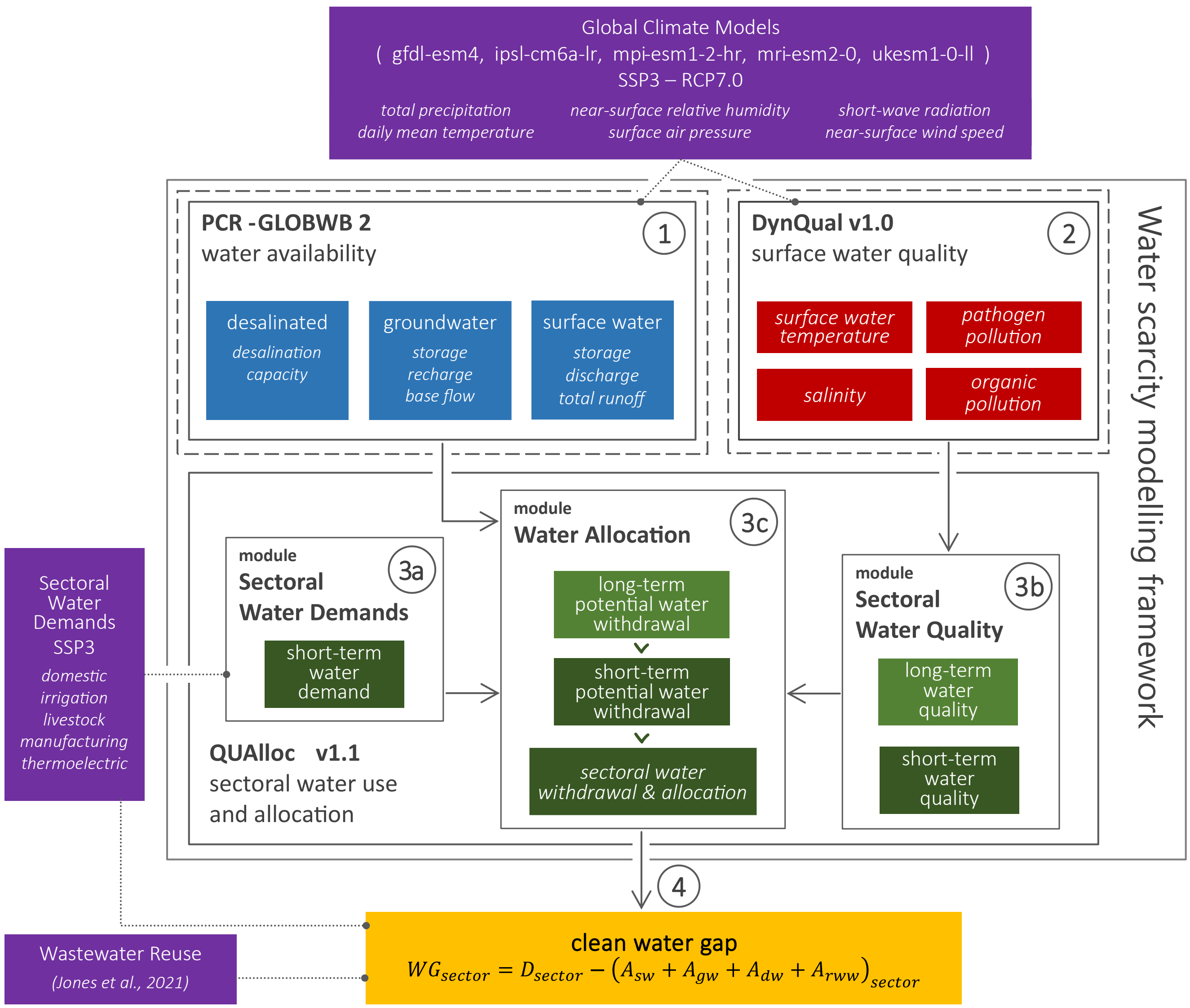

Supplementary Fig. 1 Water scarcity modelling framework: interaction between QUAlloc v1.1, PCR-GLOBWB 2, and DynQual v1.0.

Solid-line boxes within the Water Scarcity Modelling Framework represent the models (PCR-GLOBWB 2, DynQual v1.0, and QUAlloc v1.1) and the internal modules/sub-modules of QUAlloc v1.1. Dashed boxes indicate processes (i.e., model runs for PCR-GLOBWB 2 and DynQual v1.0) performed prior to the water allocation process. Purple boxes denote input data used by the models and for clean water gap estimation; blue boxes represent water availability data; red boxes represent water quality data; and green boxes represent water allocation data (light green for long-term and dark green for short-term estimates). Arrowed connections indicate modelled data transfers, whereas dotted connections indicate the transfer of input data. A description of the processes (circled numbers) is provided in Supplementary Text 1.

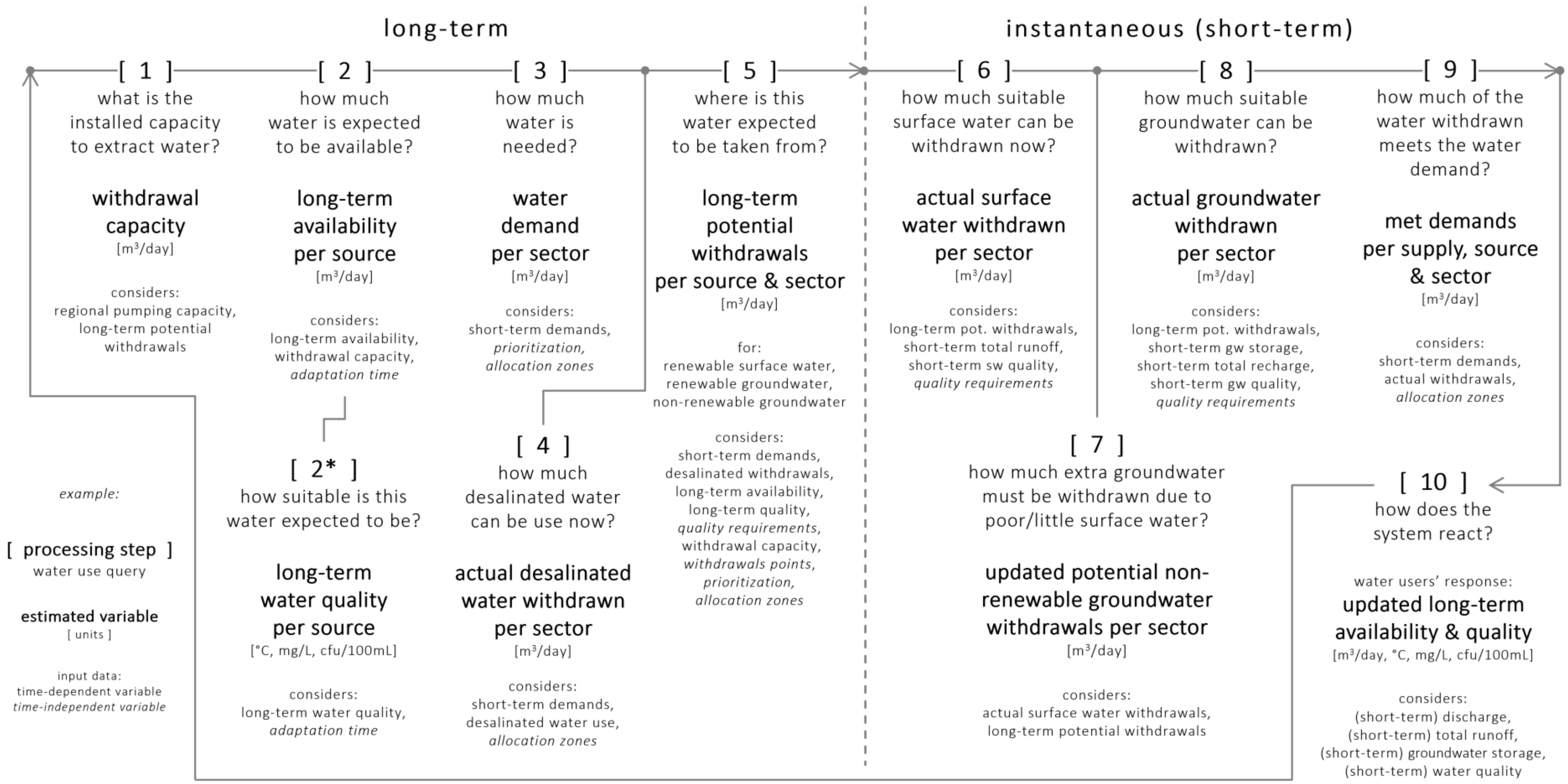

Supplementary Fig. 2 Workflow of the water use and allocation model QUAlloc v1.1, per time-step.
A detailed description of the processes (numbers in squared brackets) is provided in Supplementary Text 2.

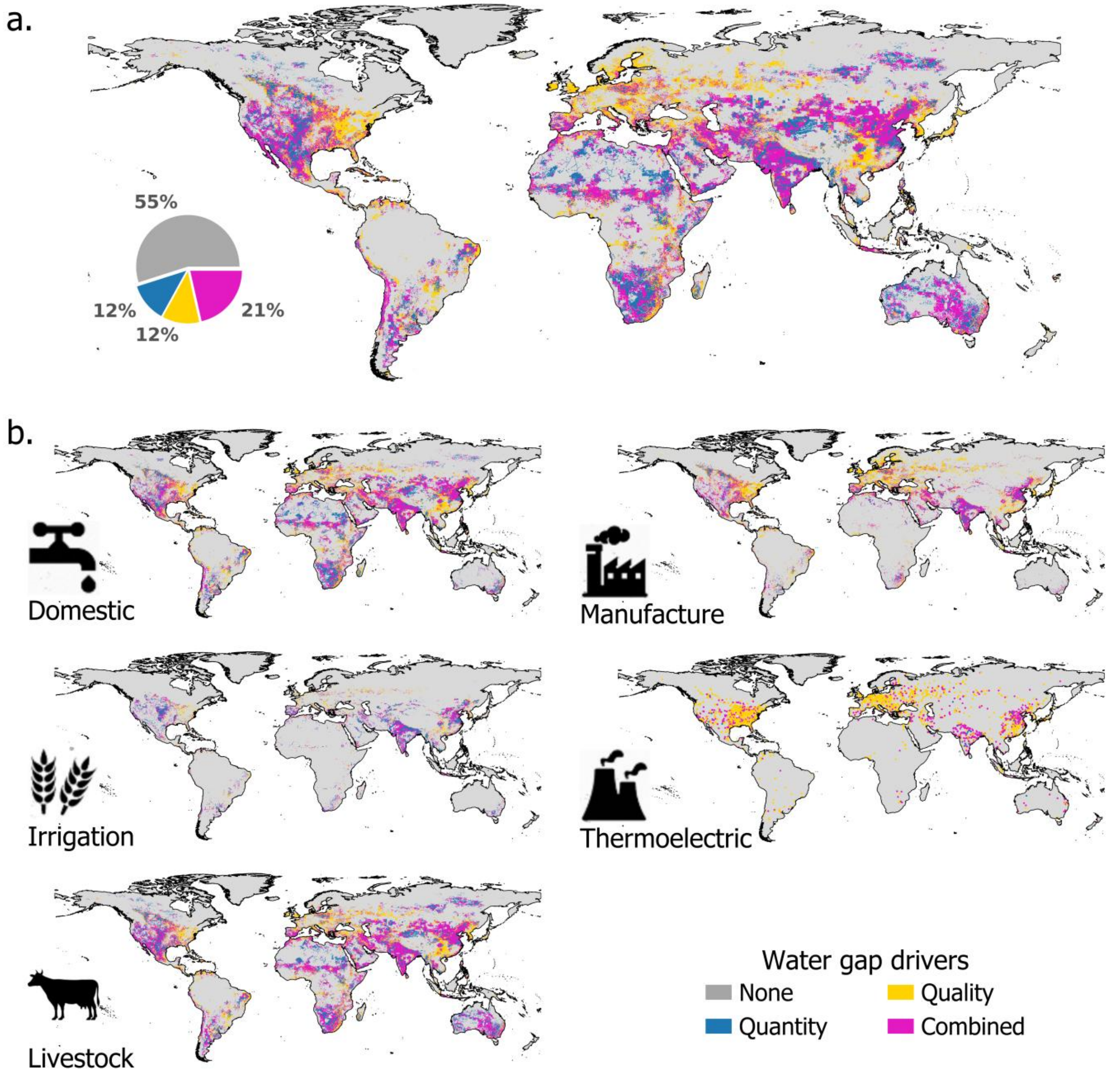

Supplementary Fig. 3 Recent past water gap drivers: water quantity, water quality and their combined effect (2005-2020). Results correspond to the ensemble mean of the simulations using the bias-corrected output of five GCMs for the global change scenario SSP3-RCP7.0. Displayed drivers are the most recurrent annual water gap driver over the period 2005-2020 over each grid-cell. Panel (a) total water gap (i.e., sum of all sectoral water gaps) and (b) per sector (i.e., domestic, irrigation, livestock, manufacturing, and thermoelectric).

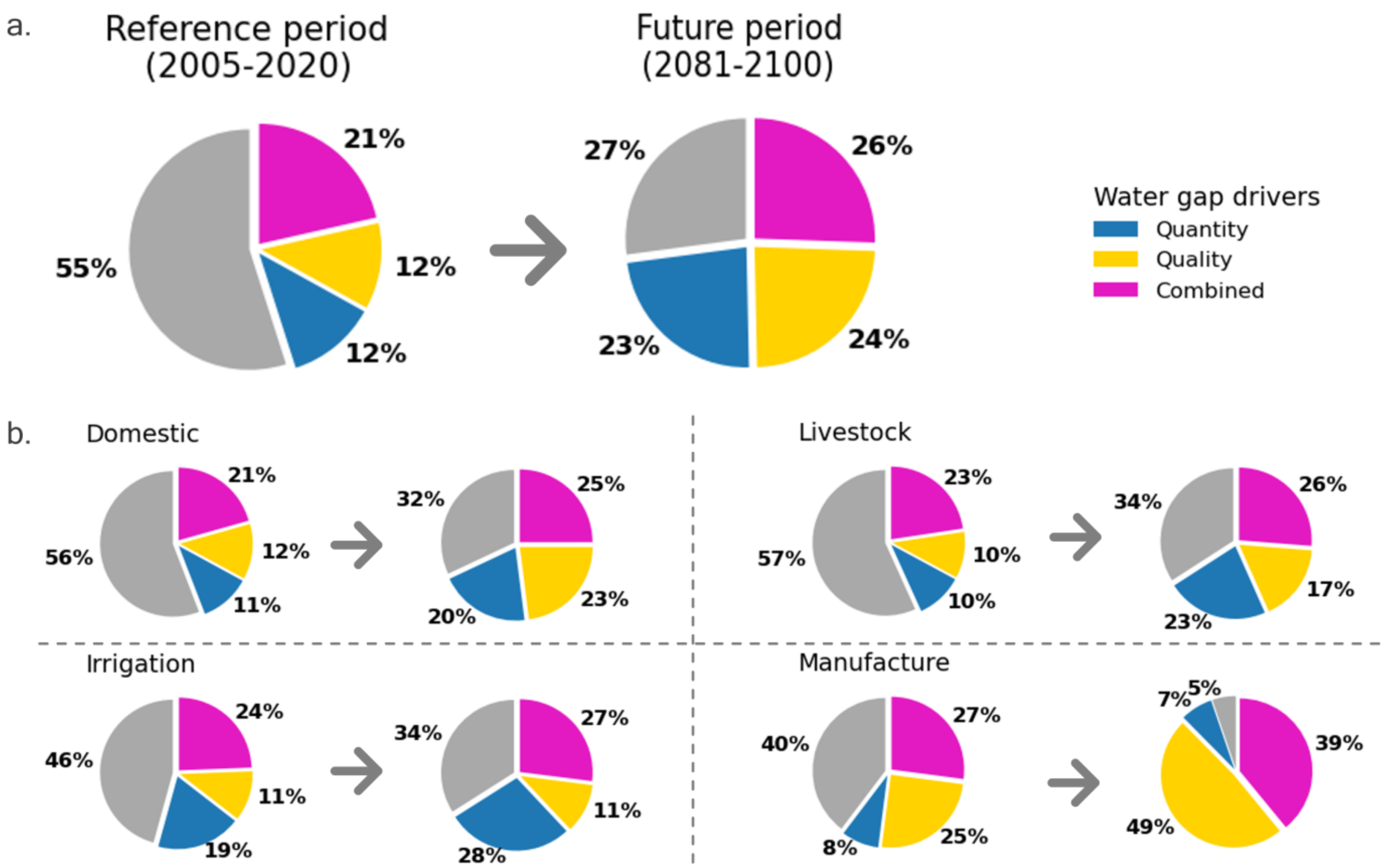

Supplementary Fig. 4 Change in the percentage of inhabited areas affected by water gap drivers: water quantity, water quality and their combined effect (past versus future).

Left pie charts correspond to reference period (2005-2020) conditions while right pie charts correspond to end of the century (2081-2100) conditions. Results correspond to the ensemble of the simulations using the bias-corrected output of five GCMs for the global change scenario SSP3-RCP7.0. Displayed drivers are the aggregation of the most recurrent water gap driver over the reference period (2005-2020) and the future period (2081-2100) over each grid-cell. Panel (a) total water gap (i.e., sum of all sectoral water gaps) and (b) per sector (i.e., domestic, irrigation, livestock, and manufacturing). Thermoelectric sector is not included since their area of influence is power plant based.

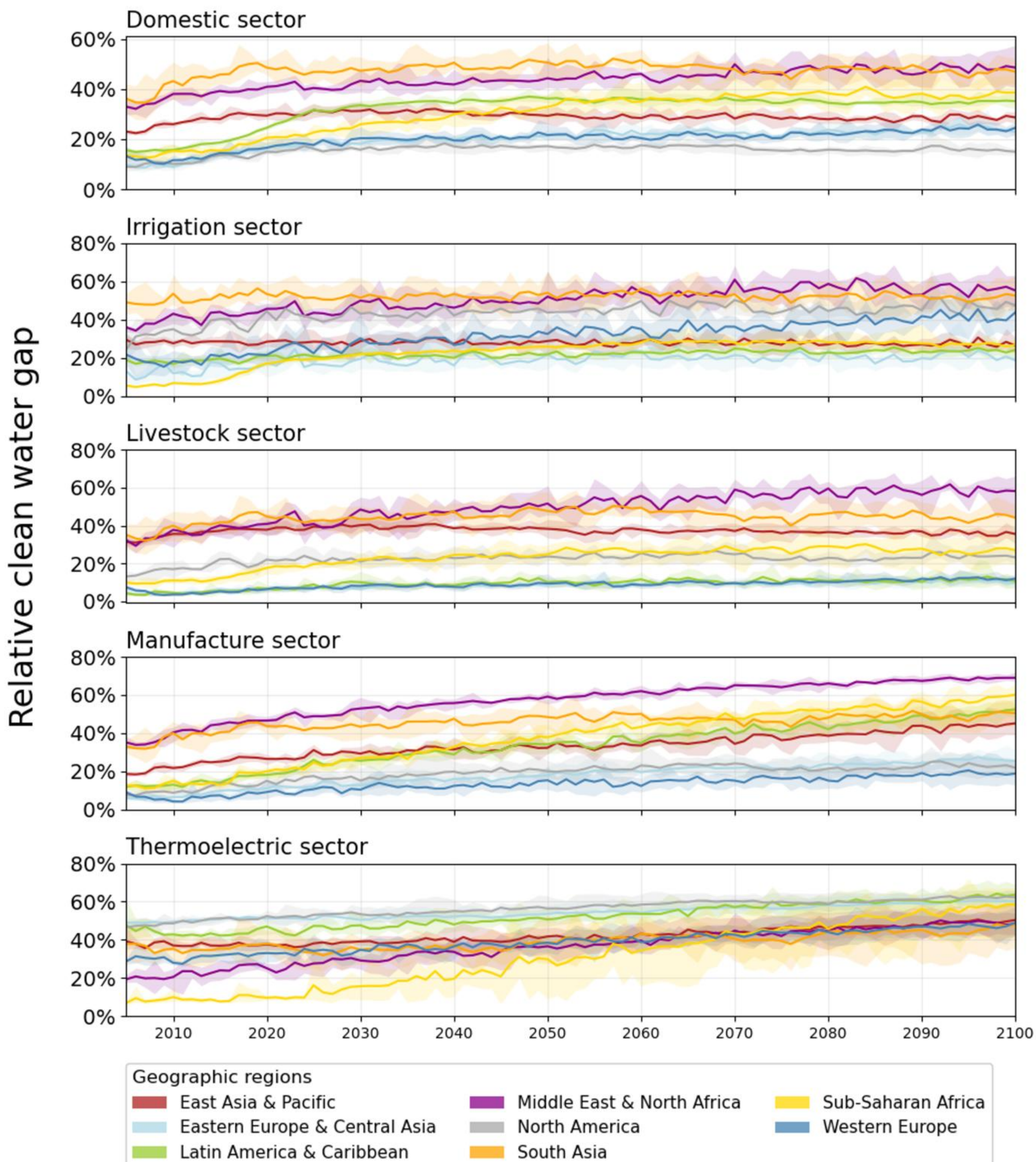

Supplementary Fig. 5 Relative clean water gap over the period 2005-2100, per geographic region and sector: domestic, irrigation, livestock, manufacturing, and thermoelectric.

Displayed results correspond to the ensemble (solid line) and the range of variation (minimum and maximum values) of their corresponding simulations using the bias-corrected output of five GCMs (coloured shades) for the global change scenario SSP3-RCP7.0 when sector-specific water quality requirements are considered, aggregated at a yearly scale.

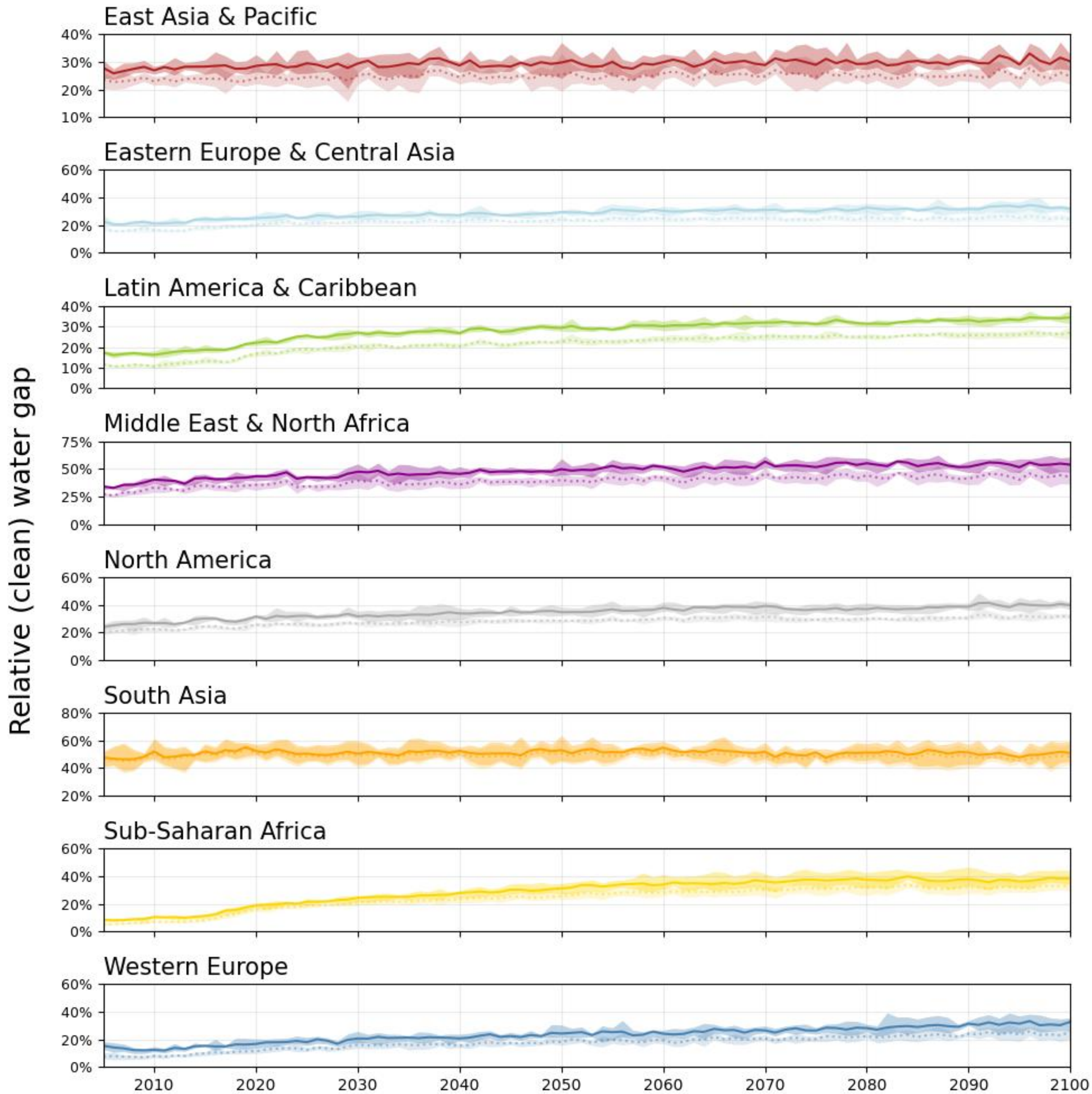

Supplementary Fig. 6 Relative (clean) water gap per geographic region over the period 2005-2100. Displayed results correspond to the ensemble (middle line) and the range of variation (minimum and maximum values) of their corresponding simulation using the bias-corrected output of five GCMs (coloured shade) for the global change scenario SSP3-RCP7.0. Solid middle line corresponds to a simulation considering water quality requirements (i.e., clean water gap), while the dotted middle line represents the simulation that disregards such water quality requirements (i.e., water gap). Results are aggregated at the yearly scale.

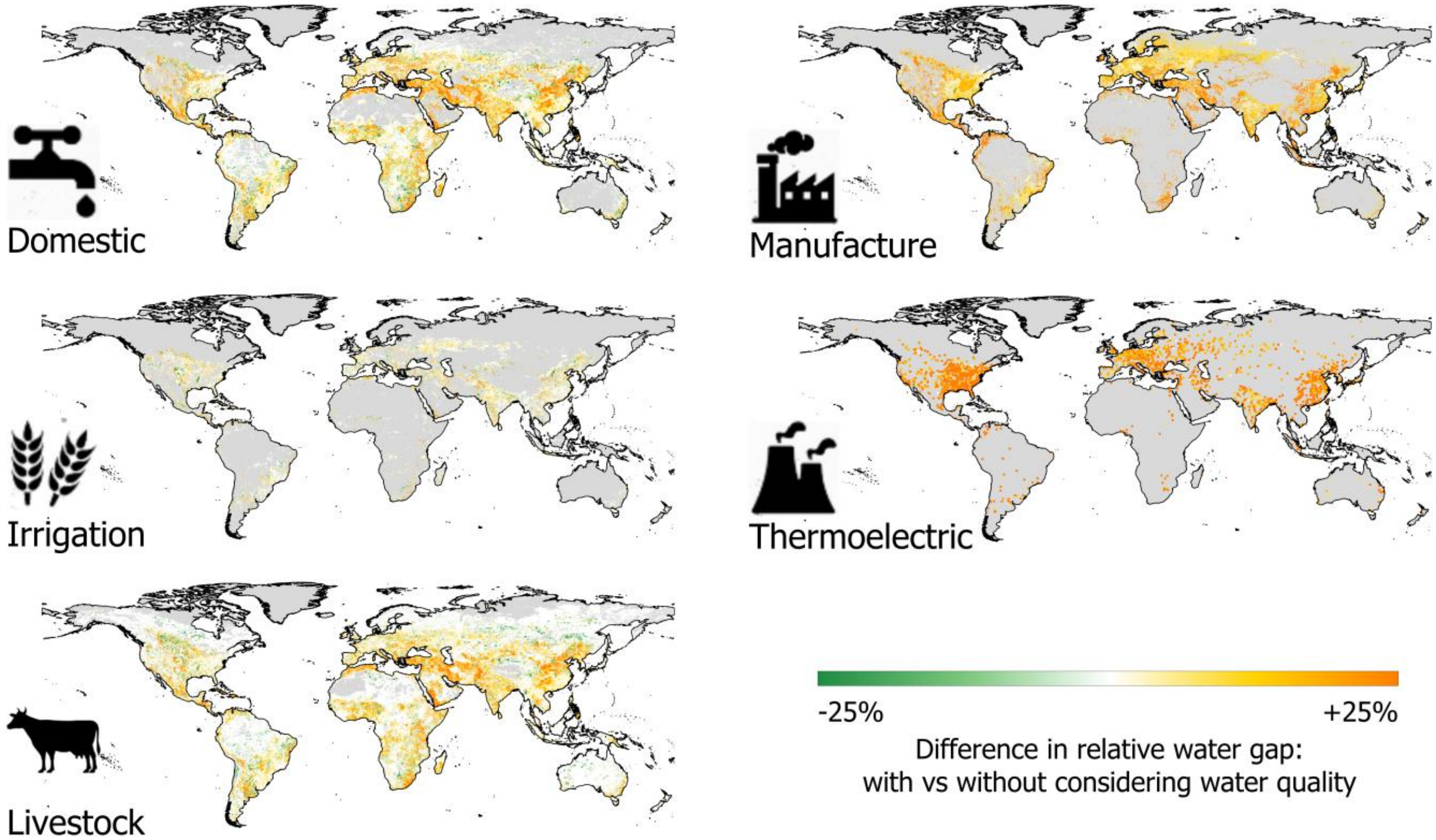

Supplementary Fig. 7 Difference in sectoral relative (clean) water gap: considering versus not sectoral water quality requirements, for the period 2081-2100.

Orange coloured locations (grid-cells) imply a relative clean water gap increase when considering water quality requirements, while green coloured locations imply the contrary. Results displayed correspond to the ensemble of the five bias-corrected GCMs for the global change scenario SSP3-RCP7.0.

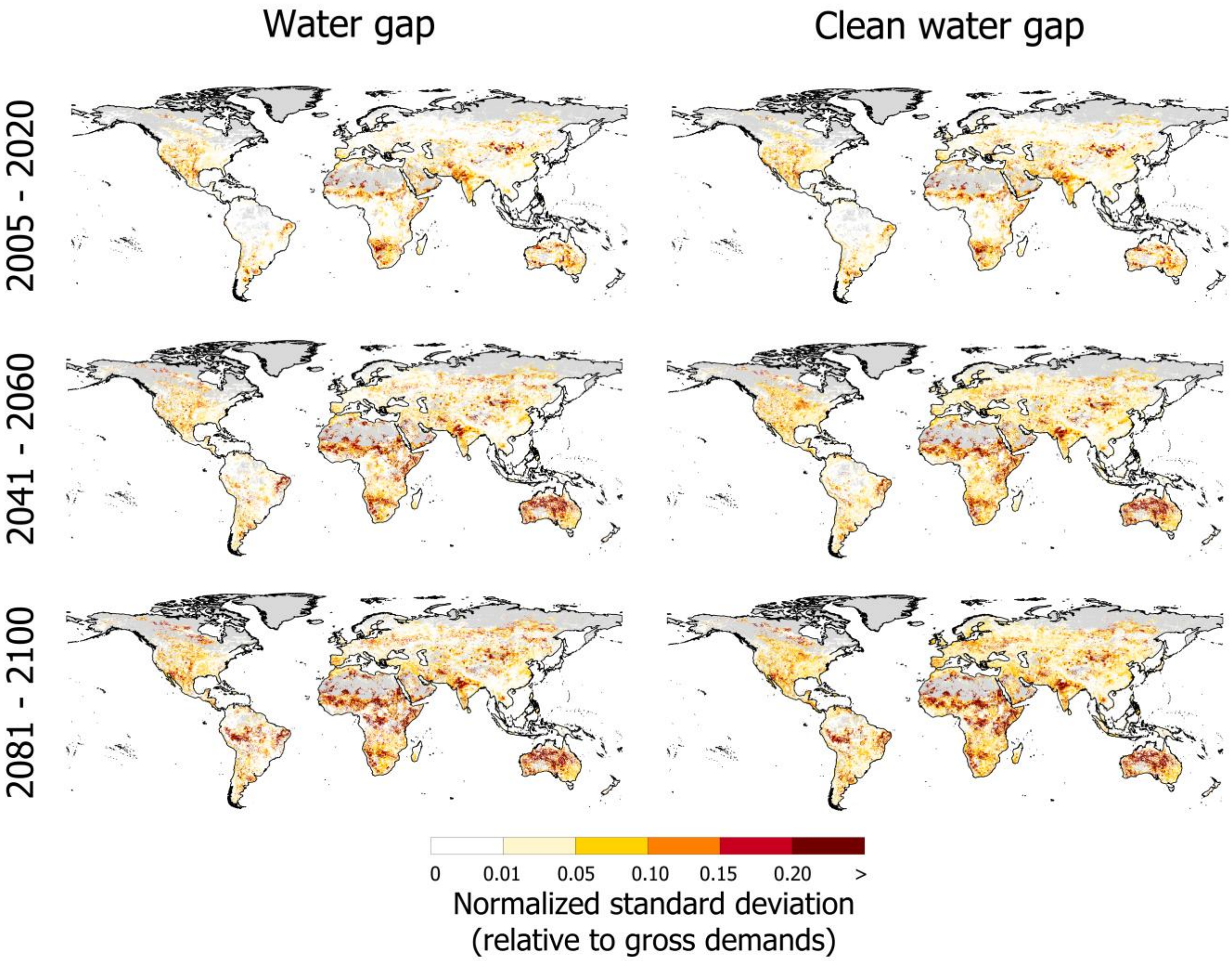

Supplementary Fig. 8 Sensitivity analysis of the influence of Global Climate Models on projected (clean) water gaps. GCMs comprise the five bias-corrected models gfdl-esm4, ipsl-cm6a-lr, mri-esm2-0, mpi-esm1-2-hr, ukesm1-0-ll, under the climate change scenario SSP3-RCP7.0. Results are standard deviation of the (clean) water gaps obtained for each GCM relative to the water demands. Results are calculated from the mean yearly (clean) water gaps over the periods of analysis. Larger values indicate more uncertainty in the results.

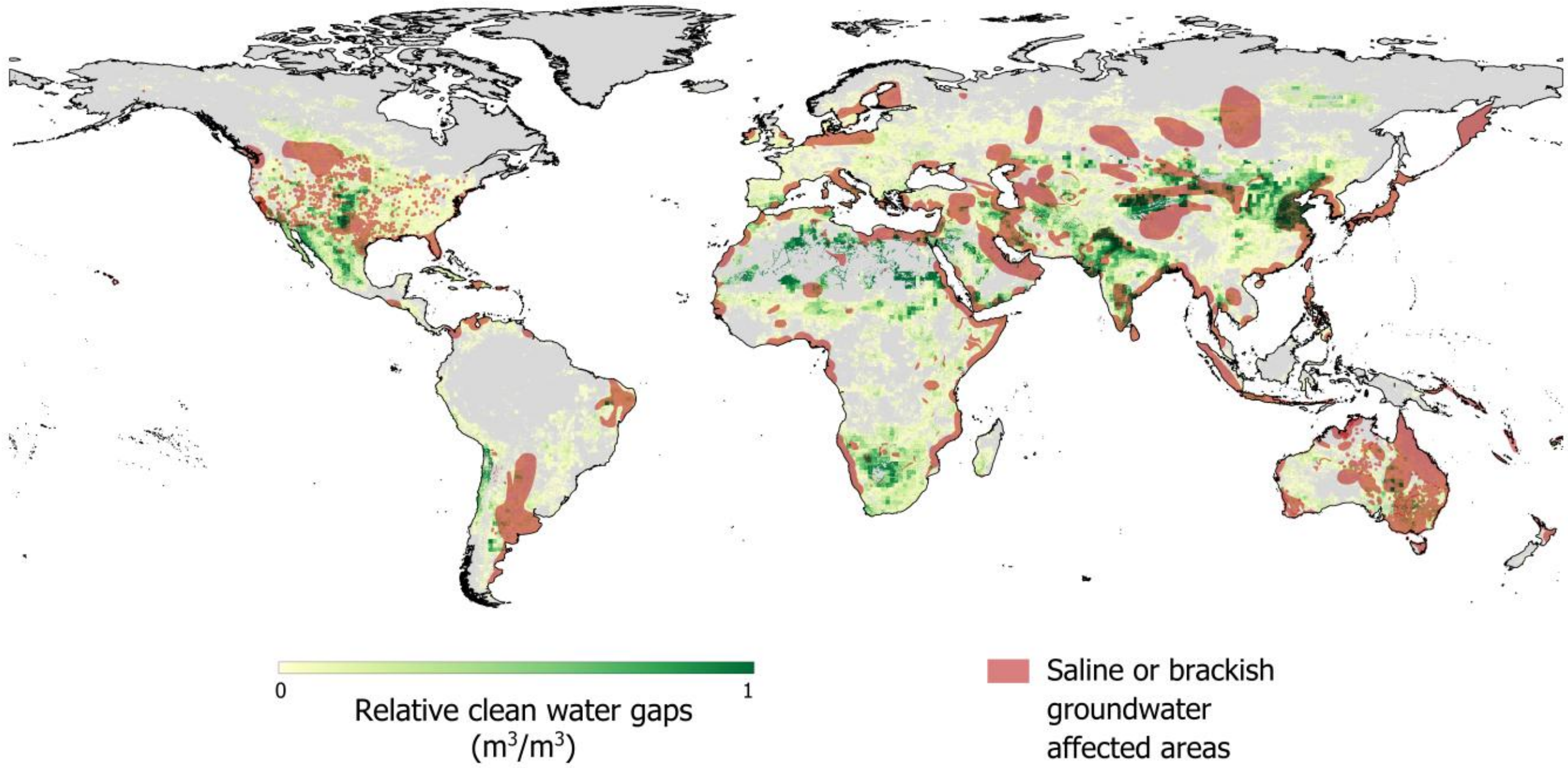

Supplementary Fig. 9 Areas affected by groundwater salinity pollution compared with relative clean water gaps during the reference period (2005-2020).

Relative clean water gaps refer to the water gaps relative to their correspondent water demand. Results represent the ensemble mean of the total relative clean water gaps (i.e., aggregated across all sectors) at annual resolution and averaged over 2005-2020. Areas shown in red indicate regions affected by saline or brackish groundwater, based on combined information from IGRAC[8] and Thorslund et al. (2020)[10]. For visualization purposes, coastal zones and well locations were adjusted: coastlines were buffered by 1.5 arc-degrees and wells by 0.5 arc-degrees. Regions identified through well-based observations[10] present a median electric conductivity higher than 3125 µS/cm (equivalent to approximately 2000 mg/L of salinity[11]), which corresponds to the water quality requirements of the most sensitive sectors: domestic and livestock (Supplementary Table 1).

Supplementary Table 1 Water quality requirements per water use sector and water quality constituent.

Water quality constituent	Units	Domestic	Irrigation	Livestock	Manufacturing	Thermoelectric
Biochemical oxygen demand	mg/L	5	30	8	15	15
Total dissolved solids	mg/L	2000	2100	2000	7000	7000
Faecal coliform	cfu/100mL	1000	5000	2000	5000	5000
Water temperature	°C	--	--	--	Grid-specific (95th percentile)	Grid-specific (95th percentile)

Values correspond to water quality standards that ensure the optimal development of the sectoral activities (i.e., avoiding salt stress by crops, cooling purposes, etc.) and to safeguard human and environmental health. Water quality standards presented comprise extensive literature research[11–18] and are used in related research[19–21]. For thermoelectric and manufacturing cooling purposes, we calculate grid-specific values of maximum permissible water temperature equivalent to the 95th percentile of daily water temperature simulation (DynQual v1.0). This methodology is based on previous research[22] and is aimed to support ecosystem health.

Supplementary Table 2 Input datasets used in QUAlloc v1.1 for analysis of future sectoral water use responses when considering water quality requirements under the global change scenario SSP3 RCP7.0.

<table>
<tr><th>Nº</th><th>Variable</th><th>Dataset</th><th>Type</th></tr>
<tr><td colspan="4">Water management</td></tr>
<tr><td>1</td><td>surface water discharge</td><td rowspan="3">PCR-GLOBWB 2 output: pseudo-naturalized</td><td rowspan="3">long-term</td></tr>
<tr><td>2</td><td>surface water runoff</td></tr>
<tr><td>3</td><td>groundwater storage</td></tr>
<tr><td>4</td><td>desalinated water production</td><td>DesalData (GWI, 2018)[7]</td><td>forcing</td></tr>
<tr><td>5</td><td>groundwater pumping capacity</td><td>PCR-GLOBWB 2 input[23]</td><td>forcing</td></tr>
<tr><td>6</td><td>wastewater reused</td><td>Jones et al. (2021)[6]</td><td>post-processing</td></tr>
<tr><td colspan="4">Water demand</td></tr>
<tr><td>7</td><td>domestic gross and net demand</td><td rowspan="3">PCR-GLOBWB 2 input[23]</td><td rowspan="5">forcing</td></tr>
<tr><td>8</td><td>livestock gross and net demand</td></tr>
<tr><td>9</td><td>manufacturing gross and net demand</td></tr>
<tr><td>10</td><td>irrigation gross demand</td><td>PCR-GLOBWB 2 output: pseudo-naturalized</td></tr>
<tr><td>11</td><td>thermoelectric gross and net demand</td><td>based on Lohrmann et al. (2019)[24], Graham et al. (2018)[25]</td></tr>
<tr><td colspan="4">Water quality</td></tr>
<tr><td>12</td><td>surface water temperature</td><td rowspan="4">DynQual v1.0 output: non-naturalized</td><td rowspan="4">forcing & long-term</td></tr>
<tr><td>13</td><td>surface water organic pollution</td></tr>
<tr><td>14</td><td>surface water salinity</td></tr>
<tr><td>15</td><td>surface water pathogen pollution</td></tr>
<tr><td colspan="4">Surface water</td></tr>
<tr><td>16</td><td>precipitation</td><td>ISIMIP 3b[4]</td><td>forcing</td></tr>
<tr><td>17</td><td>reference potential evapotranspiration</td><td rowspan="4">PCR-GLOBWB 2 output: pseudo-naturalized</td><td rowspan="3">forcing</td></tr>
<tr><td>18</td><td>direct runoff</td></tr>
<tr><td>19</td><td>interflow</td></tr>
<tr><td>20</td><td>channel storage</td><td>initial condition</td></tr>
<tr><td>21</td><td>return flow</td><td>PCR-GLOBWB 2 output: non-naturalized</td><td>initial condition</td></tr>
<tr><td colspan="4">Groundwater</td></tr>
<tr><td>22</td><td>groundwater recharge</td><td rowspan="3">PCR-GLOBWB 2 output: pseudo-naturalized</td><td>forcing</td></tr>
<tr><td>23</td><td>base flow</td><td>initial condition</td></tr>
<tr><td>24</td><td>groundwater storage</td><td>initial condition</td></tr>
</table>

All datasets included (except for **Water demands**, *desalinated water production*, *groundwater pumping capacity* and *wastewater reuse*) comprise the corresponding five GCM under evaluation: gfdl-esm4, ipsl-cm6a-lr, mpi-esm1-2-hr, mri-esm2-0, ukesm1-0-ll[4]. All datasets are at the global-scale, 5 arcmin gridded and at a monthly time-step, except for the *groundwater pumping capacity* dataset which is at a yearly time-step. **Forcing** data type covers the period 2000-2100. **Long-term** data type corresponds to the multi-annual monthly mean values for the period 2000-2015. **Initial condition** data type represents the state of the variable the day before the start of the simulation (i.e., 31/December/1999). **Post-processing** data type corresponds to datasets used on the outputs from the modelling framework. **PCR-GLOBWB 2**/**DynQual v1.0 output** data correspond to the resulting datasets of each model simulation using the GCM, under pseudo- or non-naturalized conditions, respectively. A **pseudo-naturalized** run type refers to a simulation where human water use is disregarded, thus representing the potential water readily available to be used, in contrast to a **non-naturalized** run where conditions like irrigation demands and return flow are considered and further reported.

Supplementary Table 3 Comparison of water scarcity assessments: inputs, models, criteria.

References	Models	Scenario	Climate forcing	Spatial res.	Criteria
Mekonnen (2016)[26]	Composite Runoff v1.0[27]	Historical	--	30 arcmin	WS = blue water footprint<br>WS at lest 1 month/year<br>WS > 2
Kummu (2016)[28]	WaterGAP 2[29]	Historical	WATCH[30]	Food production units	stress = consumption / availability > 0.4<br>shortage = availability / population < 1000 m^3/population/year
van Vliet (2021)[31]	PCR-GLOBWB 2[1]<br>WaterGAP 2<br>H08[32]<br>VIC[33], QUAL[31]	Historical (HYDE 3.1)[34]	WFDEI[35]	30 arcmin	$WSq_{desal+wwr}$ = includes water quality, desalination and treated wastewater reuse<br>constituents: BOD, TDS, TN, TP<br>$WSq_{desal+ww}$r > 0.4
Jones (2024)[5]	PCR-GLOBWB 2<br>DynQual v1.0	SSP3-RCP7.0	Ensemble[4]:<br>gfdl-esm4, ukesm1-0-ll, mpi-esm1-2-hr, ipsl-cm6a-lr, mri-esm2-0	5 arcmin	WSq = water scarcity including water quality<br>constituents: Tw, BOD, TDS, FC<br>WSq at least 1 month/year<br>WSq > 1
Wang (2024)[36]	MARINA-Nutrients[37]<br>MAgPIE[38]<br>VIC	Average:<br>SSP1-RCP2.6<br>SSP2-RCP2.6<br>SSP5-RCP8.5	Based on:<br>gfdl-esm2m, ipsl-cm5a-lr, miroc-esm-chem, HadGEM2-es, NorESM1-m	Basin	CWS = clean water scarcity (combines WS and TDN requirements)<br>constituents: TDN<br>WS > 0.2 & TDN > 1
Arnell (2014)[39]	MacPDM.09[40,41]	Average:<br>SSP3-RCP6.0<br>SSP3-RCP8.5	Ensemble:<br>19 CMIP5 GCMs[42]	Basin	temporal scale: annual average<br>runoff/population < 1000 m^3/population/year
Hanasaki (2013)[43]	H08	Average:<br>SSP3-RCP6.0<br>SSP3-RCP8.5	Based on:<br>gfdl-esm2m, miroc-esm-chem, HadGEM2-es	30 arcmin	CAD = Cumulative Abstraction to Demand ratio<br>CAD = cumulative abstractions / cumulative demand over a year<br>CAD < 0.5
UN (2018)[44]	PCR-GLOBWB 2<br>WaterGAP 2<br>H08	Average:<br>SSP1-RCP4.5<br>SSP2-RCP6.0<br>SSP3-RCP6.0	Based on:<br>gfdl-esm2m, ipsl-cm5a-lr, miroc-esm-chem, HadGEM2-es, NorESM1-m	30 arcmin	WS = water scarcity<br>WS at least 1 month/year<br>WS = withdrawal / surface water availability<br>WS > 0.4
He (2021)[45]	AQUEDUCT3.0[46]<br>NIER dataset[47]	Average:<br>SSP1-RCP2.6<br>SSP2-RCP4.5<br>SSP3-RCP7.0<br>SSP5-RCP8.5	Ensemble:<br>10 CMIP6 GCMs	30 arcmin	WSI = water scarcity index<br>WSI = withdrawals / availability<br>WSI > 1<br>only urban population evaluated

Where: CIMP = Coupled Model Intercomparison Project, GCM = Global Climate Model, Tw = surface water temperature, BOD = biochemical oxygen demand, TDS = total dissolved solids, FC = faecal coliforms, TDN = total dissolved nitrogen, TN = total nitrogen, TP = total phosphorus

References

1. Sutanudjaja, E. H. *et al.* PCR-GLOBWB 2: A 5 arcmin global hydrological and water resources model. *Geosci. Model Dev.* **11**, 2429–2453 (2018).
2. Jones, E. R. *et al.* DynQual v1.0: a high-resolution global surface water quality model. *Geosci. Model Dev.* **16**, 4481–4500 (2023).
3. Cárdenas Belleza, G. A., van Beek, L. P. H., Bierkens, M. F. P., Marinelli, B. P. P. & van Vliet, M. T. H. Water Quality Affects Water Source Allocation and Competition Between Sectors Globally. *Earths Future* **14**, (2026).
4. Lange, S. & Büchner, M. Secondary ISIMIP3b bias-adjusted atmospheric climate input data (v1.1). *ISIMIP Repository* (2022) doi:10.48364/ISIMIP.581124.1.
5. Jones, E. R., Bierkens, M. F. P. & van Vliet, M. T. H. Current and future global water scarcity intensifies when accounting for surface water quality. *Nat. Clim. Chang.* **14**, 629–635 (2024).
6. Jones, E. R., van Vliet, M. T. H., Qadir, M. & Bierkens, M. F. P. Country-level and gridded estimates of wastewater production, collection, treatment and reuse. *Earth Syst. Sci. Data* **13**, 237–254 (2021).
7. Global Water Intelligence. GWI / DESALDATA. https://www.desaldata.com/ (2018).
8. IGRAC. The Global Groundwater Information System (GGIS). https://ggis.un-igrac.org/ (2025).
9. Robinson, S. *et al. The International Model for Policy Analysis of Agricultural Commodities and Trade (IMPACT): Model Description for Version 3*. (2015).
10. Thorslund, J. & van Vliet, M. T. H. A global dataset of surface water and groundwater salinity measurements from 1980–2019. *Sci. Data* **7**, 1–11 (2020).
11. Ayers, R. S. & Westcot, D. W. *Water Quality for Agriculture. FAO Irrigation and Drainage*. *FAO Irrigation and Drainage Paper* vol. No.29 (1985).
12. UNEP. *A Snapshot of the World's Water Quality: Towards a Global Assessment*. *United Nations Environment Programme* (2016).
13. WHO. *Guidelines for Drinking-Water Quality: Fourth Edition Incorporating the First Addendum*. (World Health Organization, Geneva, Switzerland, 2017).
14. EPA. *Guidelines for Water Reuse*. (2004).
15. Salgot, M., Huertas, E. & Dott, W. *Guideline for Quality Standards for Water Reuse in Europe*. https://www.researchgate.net/publication/233988054 (2006).
16. Fipps, G. *Irrigation Water Quality Standards and Salinity Management Strategies*. (2003).
17. Zaman, M., Shahidd, S. A. & Heng, L. *Guideline for Salinity Assessment, Mitigation and Adaptation Using Nuclear and Related Techniques*. (2018) doi:10.1007/978-3-319-96190-3.
18. Harto, C., Finster, M., Schroeder, J. & Clark, C. *Saline Water for Power Plant Cooling - Challenges and Opportunities*. (2014).
19. van Vliet, M. T. H., Flörke, M. & Wada, Y. Quality matters for water scarcity. *Nat. Geosci.* **10**, 800–802 (2017).
20. Jones, E. R. *et al.* Current wastewater treatment targets are insufficient to protect surface water quality. *Commun. Earth Environ.* **3**, (2022).
21. Jones, E. R. *et al.* Sub-Saharan Africa will increasingly become the dominant hotspot of surface water pollution. *Nature Water* **1**, 602–613 (2023).
22. van Vliet, M. T. H., Wiberg, D., Leduc, S. & Riahi, K. Power-generation system vulnerability and adaptation to changes in climate and water resources. *Nat. Clim. Chang.* **6**, 375–380 (2016).
23. Sutanudjaja, E. *et al.* PCR-GLOBWB 2 input files version 2017_11_beta_1. Preprint at https://doi.org/10.5281/zenodo.1045339 (2017).
24. Lohrmann, A., Farfan, J., Caldera, U., Lohrmann, C. & Breyer, C. Global scenarios for significant water reduction in thermal power plants based on cooling water demand estimation using satellite imagery. *Nat. Energy* **4**, (2019).
25. Graham, N. T. *et al.* Water Sector Assumptions for the Shared Socioeconomic Pathways in an Integrated Modeling Framework. *Water Resour. Res.* **54**, 6423–6440 (2018).
26. Mekonnen, M. M. & Hoekstra, A. Y. Sustainability: Four billion people facing severe water scarcity. *Sci. Adv.* **2**, 1–7 (2016).
27. Fekete, B. M., Vörösmarty, C. J. & Grabs, W. High-resolution fields of global runoff combining observed river discharge and simulated water balances. *Global Biogeochem. Cycles* **16**, 15-1-15–10 (2002).

28. Kummu, M. *et al.* The world's road to water scarcity: Shortage and stress in the 20th century and pathways towards sustainability. *Sci. Rep.* **6**, (2016).
29. Alcamo, J. *et al.* Development and testing of the WaterGAP 2 global model of water use and availability. *Hydrological Sciences Journal* **48**, 317–337 (2003).
30. Weedon, G. P. *et al.* Creation of the WATCH forcing data and its use to assess global and regional reference crop evaporation over land during the twentieth century. *J. Hydrometeorol.* **12**, 823–848 (2011).
31. van Vliet, M. T. H. *et al.* Global water scarcity including surface water quality and expansions of clean water technologies. *Environmental Research Letters* **16**, (2021).
32. Hanasaki, N. *et al.* An integrated model for the assessment of global water resources - Part 1: Model description and input meteorological forcing. *Hydrol. Earth Syst. Sci.* **12**, 1007–1025 (2008).
33. Droppers, B., Franssen, W. H. P., van Vliet, M. T. H., Nijssen, B. & Ludwig, F. Simulating human impacts on global water resources using VIC-5. *Geosci. Model Dev.* **13**, 5029–5052 (2020).
34. Klein Goldewijk, K., Beusen, A., Doelman, J. & Stehfest, E. Anthropogenic land use estimates for the Holocene - HYDE 3.2. *Earth Syst. Sci. Data* **9**, 927–953 (2017).
35. Weedon, G. P. *et al.* The WFDEI meteorological forcing data set: WATCH Forcing Data methodology applied to ERA-Interim reanalysis data. *Water Resour. Res.* **50**, 7505–7514 (2014).
36. Wang, M. *et al.* A triple increase in global river basins with water scarcity due to future pollution. *Nat. Commun.* **15**, (2024).
37. Strokal, M. *et al.* Urbanization: an increasing source of multiple pollutants to rivers in the 21st century. *npj Urban Sustainability* **1**, (2021).
38. Dietrich, J. P. *et al.* MAgPIE 4-a modular open-source framework for modeling global land systems. *Geosci. Model Dev.* **12**, 1299–1317 (2019).
39. Arnell, N. W. & Lloyd-Hughes, B. The global-scale impacts of climate change on water resources and flooding under new climate and socio-economic scenarios. *Clim. Change* **122**, 127–140 (2014).
40. Gosling, S. N. & Arnell, N. W. Simulating current global river runoff with a global hydrological model: Model revisions, validation, and sensitivity analysis. *Hydrol. Process.* **25**, 1129–1145 (2011).
41. Arnell, N. W. & Gosling, S. N. The impacts of climate change on river flow regimes at the global scale. *J. Hydrol. (Amst).* **486**, 351–364 (2013).
42. Taylor, K. E., Stouffer, R. J. & Meehl, G. A. An overview of CMIP5 and the experiment design. *Bulletin of the American Meteorological Society* vol. 93 485–498 Preprint at https://doi.org/10.1175/BAMS-D-11-00094.1 (2012).
43. Hanasaki, N. *et al.* A global water scarcity assessment under Shared Socio-economic Pathways - Part 2: Water availability and scarcity. *Hydrol. Earth Syst. Sci.* **17**, 2393–2413 (2013).
44. World Water Assessment Programme. *The United Nations World Water Development Report 2018 - Nature-Based Solutions for Water*. (2018).
45. He, C. *et al.* Future global urban water scarcity and potential solutions. *Nat. Commun.* **12**, (2021).
46. Hofsté, R. W. *et al. AQUEDUCT 3.0: Updated Decision-Relevant Global Water Risk Indicators*. https://www.wri.org/publication/aqueduct-30 (2019).
47. Hanasaki, N. *et al.* A global water scarcity assessment under Shared Socio-economic Pathways - Part 1: Water use. *Hydrol. Earth Syst. Sci.* **17**, 2375–2391 (2013).
